# Long noncoding RNA DLGAP1-AS2 promotes tumorigenesis and metastasis by regulating the Trim21/ELOA/LHPP axis in colorectal cancer

**DOI:** 10.1186/s12943-022-01675-w

**Published:** 2022-11-14

**Authors:** Xue Wang, Han Cheng, Jing Zhao, Jiuming Li, Ying Chen, Kaisa Cui, Lu Tian, Jia Zhang, Chaoqun Li, Shengbai Sun, Yuyang Feng, Surui Yao, Zehua Bian, Shenglin Huang, Bojian Fei, Zhaohui Huang

**Affiliations:** 1 Wuxi Cancer Institute, Affiliated Hospital of Jiangnan University, 200 Hui He Road, Jiangsu 214062 Wuxi, China; 2 Laboratory of Cancer Epigenetics, Wuxi School of Medicine, Jiangnan University, 214122 Wuxi, Jiangsu China; 3 Institutes of Biomedical Sciences, Fudan University Shanghai Cancer Center, Fudan University, 200032 Shanghai, China; 4 Department of Gastrointestinal Surgery, Affiliated Hospital of Jiangnan University, 214122 Wuxi, Jiangsu China

**Keywords:** Colorectal cancer, Long noncoding RNAs, DLGAP1-AS2, ELOA, Trim21, LHPP

## Abstract

**Background:**

Long noncoding RNAs (lncRNAs) have driven research focused on their effects as oncogenes or tumor suppressors involved in carcinogenesis. However, the functions and mechanisms of most lncRNAs in colorectal cancer (CRC) remain unclear.

**Methods:**

The expression of DLGAP1-AS2 was assessed by quantitative RT-PCR in multiple CRC cohorts. The impacts of DLGAP1-AS2 on CRC growth and metastasis were evaluated by a series of in vitro and in vivo assays. Furthermore, the underlying mechanism of DLGAP1-AS2 in CRC was revealed by RNA pull down, RNA immunoprecipitation, RNA sequencing, luciferase assays, chromatin immunoprecipitation, and rescue experiments.

**Results:**

We discovered that DLGAP1-AS2 promoted CRC tumorigenesis and metastasis by physically interacting with Elongin A (ELOA) and inhibiting its protein stability by promoting tripartite motif containing 21 (Trim21)-mediated ubiquitination modification and degradation of ELOA. In particular, we revealed that DLGAP1-AS2 decreases phospholysine phosphohistidine inorganic pyrophosphate phosphatase (LHPP) expression by inhibiting ELOA-mediated transcriptional activating of LHPP and thus blocking LHPP-dependent suppression of the AKT signaling pathway. In addition, we also demonstrated that DLGAP1-AS2 was bound and stabilized by cleavage and polyadenylation specificity factor (CPSF2) and cleavage stimulation factor (CSTF3).

**Conclusions:**

The discovery of DLGAP1-AS2, a promising prognostic biomarker, reveals a new dimension into the molecular pathogenesis of CRC and provides a prospective treatment target for this disease.

**Supplementary Information:**

The online version contains supplementary material available at 10.1186/s12943-022-01675-w.

## Background

Colorectal cancer (CRC) is the third most common malignant carcinoma and the second leading cause of cancer-related death worldwide. The incidence of CRC is continually increasing, and it is estimated that approximately 1.9 million new CRC cases emerged and 935,000 deaths occurred in 2020 [1]. The molecular pathogenesis of CRC has not been fully elucidated, and limited success has been achieved in improving the survival of CRC patients. Thus, constant efforts are required to elucidate the underlying molecular mechanisms and identify novel therapeutic targets.

Current progress in cancer transcriptomics has demonstrated that many cancer-related genes are noncoding RNAs (ncRNAs). As a major member of the ncRNA family, long ncRNAs (lncRNAs) have gained widespread attention. Gradually accumulating evidence has shown that lncRNAs participate in various physiological and pathological processes by regulating protein-protein, RNA-protein or protein-DNA interactions, as well as by sponging miRNAs [2]. Some lncRNAs have been shown to contribute to CRC development and could be used as biomarkers for cancer diagnostics and therapy [3–9].

We extensively analyzed the differentially expressed lncRNAs in the CRC genome and characterized a series of CRC-related lncRNAs, including FEZF1-AS1, LINC00152 ( CYTOR ), MCM3AP-AS1, SLCO4A1-AS1, SNHG6, SNHG15, SHNG17 and UCA1 [7–15]. For example, we showed that FEZF1-AS1 promotes CRC tumorigenesis and progression by regulating PKM2/STAT3 signaling and glycolysis [7]. In addition, we revealed that CYTOR drives CRC progression by interacting with NCL and Sam68 [12].

In this study, we identified a novel transcript of DLGAP1-AS2 that is significantly upregulated in CRC and is associated with the malignant features and prognosis of CRC. Functional and mechanistic studies revealed that DLGAP1-AS2 promotes CRC tumorigenesis and progression by enhancing Trim21-mediated ubiquitination and degradation of Elongin A (ELOA). Furthermore, ELOA directly binds to the promoter of LHPP and increases its expression, thus activating LHPP-mediated suppression of the AKT signaling pathway. In addition, we also demonstrated that cleavage and polyadenylation specificity factor (CPSF2) and cleavage stimulation factor (CSTF3) bind to DLGAP1-AS2 and synergistically increase its stability in CRC cells. Our study uncover a previously unknown regulatory axis of DLGAP1-AS2/Trim21/ELOA/LHPP in CRC and highlight that manipulation of this signaling axis may be a novel strategy for the treatment of CRC and other tumors.

## Methods

### Cell lines

The CRC cell lines Caco-2, DLD1, HCT116, HCT8, HT29, LoVo, RKO and SW480 were purchased from ATCC and cultured following their instructions. These cells were characterized by Genewiz, Inc.(China) using short tandem repeat markers and were confirmed to be mycoplasma-free.

### Clinical samples

Human primary CRC tissues and their paired adjacent noncancerous tissues (NCTs) were collected from Affiliated Hospital of Jiangnan University with informed consent. The study was approved by the Clinical Research Ethics Committees of Affiliated Hospital of Jiangnan University, and written informed consent was obtained from all patients.

### RNA-sequence analyses

The total RNA samples were purified from CRC and NCTs using TRIzol (Invitrogen, USA) and were treated with Ribo-off rRNA Depletion Kit (Vazyme, China). These treated RNA samples were subjected to RNA-seq library construction using VAHTS Total RNA-seq (H/M/R) Library Prep Kit for Illumina (Vazyme). These constructed libraries were then sequenced by Illumina sequencing platform on a 150 bp paired-end run. Sequencing reads were aligned using the spliced read aligner HISAT2, with Genome Reference Consortium GRCh38 as the reference genome. Annotations of lncRNAs and mRNAs in the human genome were retrieved from the GENCODE (v25) database. In addition, the expression profiling data and the relevant clinical information of several CRC cohorts were downloaded from TCGA (https://portal.gdc.cancer.gov/) and GEO (http://www.ncbi.nlm.nih.gov/geo).

### Quantitative RT-PCR(qRT-PCR)

Total RNA was reverse transcribed into cDNA using the HiFiScript cDNA Synthesis Kit (CWBIO, China). Gene expression levels were measured by qRT-PCR using Ultra SYBR Mixture (Vazyme, China). The relative gene expression levels were normalized to those of β-actin and calculated using the 2^−△△Ct^ method. The sequences of the related primers are listed in Tab.S1.

### Vector constructs and siRNA

The DLGAP1-AS2 sequence was cloned into the lentiviral expression vector pLenti-EF1a -F2A-Puro-CMV-MCS. The CPSF2 and ELOA sequences were cloned into the expression vector pRK7-Flag. The CSTF3 sequence or Trim21 sequence was cloned into the expression vector PCMV5-HA or pcDNA3.1-Myc, respectively. SiRNAs targeting DLGAP1-AS2, CPSF2, CSTF3, ELOA and Trim21 were purchased from GenePharma (China). The validated shRNA sequence of DLGAP1-AS2 or ELOA was synthesized and cloned into the pLKO.1 lentiviral expression vector. The promoter of LHPP was amplified from human genomic DNA by PCR and cloned into the pGL3-Basic vector. The related sequences are listed in Tab.S2-3.

### Cell proliferation and colony formation assays

Cell viability was measured with the Cell Counting Kit 8 (CCK8, Beyotime, China) according to the manufacturer’s instructions. For the colony formation assay, 800 to 1500 CRC cells were seeded into each well of a 6-well plate and maintained in medium containing 10% FBS for 10–15 days. The colonies were fixed with methanol, stained with 0.1% crystal violet and counted using an inverted microscope. Each experiment was repeated at least three times.

### Invasion and migration assays

Migration and invasion assays were performed in Transwell chambers (Corning, USA) according to the manufacturer’s instructions.

### In vivo assays

Male athymic BALB/c nude mice were purchased from the Shanghai Animal Center, Chinese Academy of Sciences and maintained under specific pathogen-free conditions at Jiangnan University. Nude mice, aged 5 weeks old, randomly divided into different groups (*n* = 5 for each group) were injected subcutaneously with 0.1 ml of a cell suspension containing 2 × 10^6^ CRC cells. The tumor size was measured, and the tumor volume was calculated according to the formula volume = length×width^2^ × 0.5. For the in vivo metastasis model, 2 × 10^6^ CRC cells were injected into 7-week-old male BALB/c nude mice randomly divided into different groups (*n* = 5 for each group) by tail vein. Five weeks after injection, the lung nodules in the mice were observed to measure the capability of the cells to form metastatic tumors. All animal experiments were performed in accordance with the relevant institutional and national guidelines and the regulations of Jiangnan University Medical Experimental Animal Care Commission (JN.No20190615b0320925).

### RNA pull-down assays and mass spectrometry analyses

RNA pull-down assays were performed using the Pierce™ Magnetic RNA-Protein Pull-Down Kit (Thermo Fisher, USA) according to the manufacturer’s instructions. The RNA pull-down samples were separated by gel electrophoresis and visualized with silver staining. Specific bands were excised for proteomics screening by mass spectrometry analyses and retrieved from the Human Protein Reference Database (http://www.hprd.org/). The primers for DLGAP1-AS2 and its deletion fragments for in vitro transcription are provided in Tab.S4.

### RNA immunoprecipitation (RIP) assays

RIP assays were performed using the Magna RIP RNA-Binding Protein Immunoprecipitation Kit (Millipore, USA) according to the manufacturer’s instructions. Cell lysates were incubated overnight at 4 °C with magnetic beads conjugated to anti-CPSF2, anti-CSTF3, anti-ELOA or anti-IgG. The RIP samples were subjected to RNA extraction and subsequent RT-PCR analyses to detect the abundance of DLGAP1-AS2. Information about the antibodies is listed in Tab.S5.

### Immunoblotting analyses

Cells were lysed in lysis buffer (Beyotime) with protease inhibitor cocktail treatment (Roche, USA). The protein extracts were then separated by SDS–PAGE and transferred to PVDF membranes (Millipore, USA). After blocking, the membranes were incubated with primary antibodies at 4 °C overnight and were then incubated with a peroxidase conjugated secondary antibody (1:5000, Thermo Fisher) for 1 h at room temperature. Finally, the membranes were visualized with ECL substrate (Vazyme).

### Immunoprecipitation (IP) assay

The indicated plasmids were transfected into HCT116 cells and lysed in IP buffer (Beyotime) containing protease inhibitors. The supernatants were incubated with the beads overnight at 4 °C with gentle rotation. The beads with the attached immune complexes were washed three times and analyzed by immunoblotting with the indicated antibodies.

### CRISPR-cas9 mediated Trim21 knock-out

Two sgRNA target DNA sequences located in exon 2 of TRIM21 were designed using the CRISPR library and inserted into pLentiCRISPR (Trim21-gRNA-pLentiCRISPR). The plasmids were transfected into HCT116 cells to generate a Trim21 knockout (KO) cell line according to the manufacturer´s instructions. Cells were treated with puromycin and separated into single cell to form colonies. Those colonies were harvested and verified for Trim21 KO by Sanger sequencing and western blotting.

### Immunohistochemistry(IHC) staining

ELOA protein levels in CRC tissues were determined by IHC. IHC staining was performed on 4-mm sections of paraffin-embedded tissue samples. Briefly, the slides were incubated with an anti-ELOA antibody (Santa, 1:200) at 4 °C overnight. The subsequent steps were performed using the GT Vision III Detection System/Mo&Rb (GeneTech, China). The slides were concurrently examined and scored by two blinded pathologists.

### Chromatin immunoprecipitation(ChIP)-on-chip arrays and data analyses

The protein-DNA complexes were precipitated using an antibody against ELOA or IgG. The ChIP samples were then amplified, labeled and hybridized to Nimblegen human 720 K RefSeq promoter arrays (Roche Nimblegen, USA). The significant peak regions with false discovery rates ≤ 0.05 were mapped to the nearest genes. The fasta sequences were extracted from the promoters of the differentially expressed genes (DEGs) from ChIP data. The MEME-ChIP database and Markov model were used to search the candidate motif sites of ELOA in the promoters of the potential ELOA target genes.

### Dual luciferase reporter assays

Luciferase reporter plasmids were co-transfected with ELOA expression plasmids. The luciferase activities of these cells were detected at 48 h after transfection using the Dual-Luciferase® Reporter Assay System (Beyotime).

### Statistical analyses

The data are presented as the mean ± standard deviation. The statistical analyses were conducted using GraphPad Prism 8.0 (GraphPad Software, USA) and SPSS 20.0 (SPSS Inc., USA), and *p* < 0.05 was considered to be statistically significant.

## Results

### Increased DLGAP1-AS2 expression is associated with poor clinical outcomes in CRC patients

**Fig. 1:**
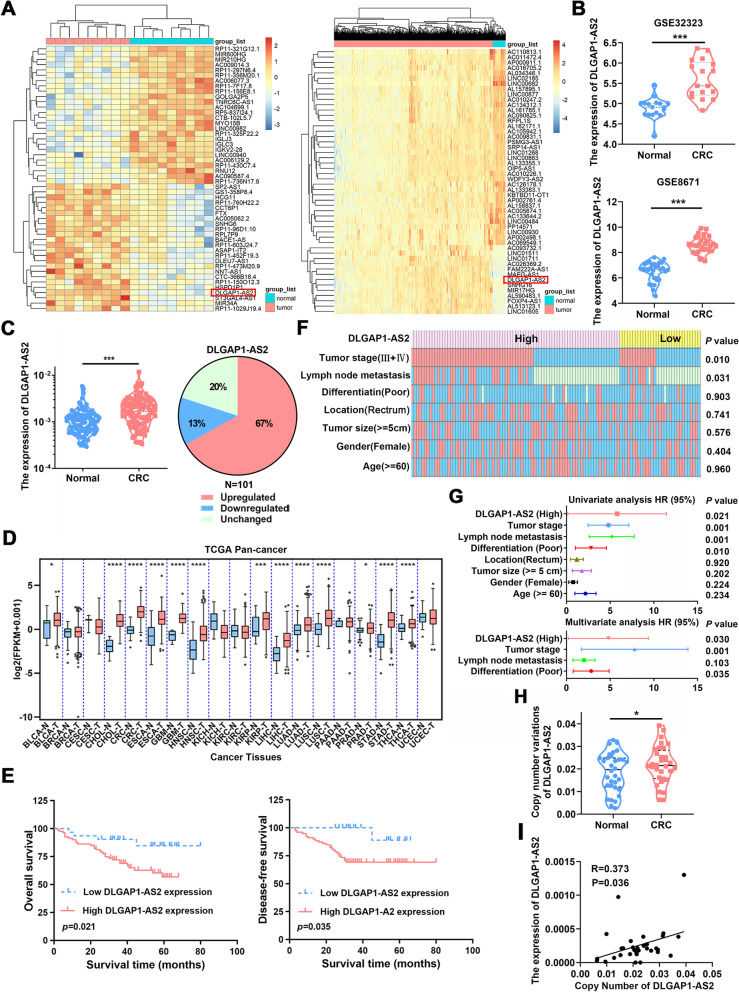
DLGAP1-AS2 is associated with a poor prognosis in patients with CRC. **A** Heatmap of significant differentially expressed lncRNAs in the RNA-seq data and TCGA CRC datasets. Transcriptional profiling analyses were performed in nine CRC and corresponding normal tissues using next-generation sequencing. **B** The expression of DLGAP1-AS2 in the CRC cohorts of GSE32323 and GSE8671. **C** The relative levels of DLGAP1-AS2 were quantified in 101 pairs of CRC tissues and adjacent normal tissues by using qRT-PCR. **D** The expression of DLGAP1-AS2 in pancancer. **E** Kaplan–Meier survival analyses of overall survival and disease-free survival according to the expression of DLGAP1-AS2 in CRC tissues. **F** The relationship between the DLGAP1-AS2 expression and clinicpathological parameters in CRC. **G** Univariate and multivariate regression analyses of CRC patients. **H** The copy number of DLGAP1-AS2 in CRC tissues detected by qPCR. **I** Correlation analyses between the copy number and expression of DLGAP1-AS2 in CRC tissues

To identify potential CRC-related lncRNAs, we performed transcriptional profiling analyses using next-generation sequencing in nine paired CRC and NCTs. The top 50 differentially expressed lncRNAs were verified with the data from the TCGA CRC cohort, and DLGAP1-AS2 showed significant upregulation in both CRC cohorts (Fig. 1 A). The aberrant upregulation of DLGAP1-AS2 was further validated in four additional CRC cohorts (GSE32323, GSE8671, GSE18105 and GSE22598; Fig. 1B and Fig.S1A). Further experimental validation in an independent CRC cohort showed that 67% (67 of 101) of cases showed more than 1.5-fold upregulation of DLGAP1-AS2 in CRCs compared with the adjacent NCTs (Fig. 1 C). Moreover, pancancer analyses revealed that DLGAP1-AS2 was highly expressed in other types of cancer, including stomach adenocarcinoma, esophageal carcinoma, pancreatic adenocarcinoma, cholangiocarcinoma and kidney renal papillary cell carcinoma, suggesting that DLGAP1-AS2 is a key cancer-related lncRNA (Fig. 1D). Thus, we focused on DLGAP1-AS2 for subsequent study.

Kaplan–Meier survival analyses showed that high DLGAP1-AS2 expression was significantly correlated with poor overall survival and disease-free survival (Fig. 1E). Correlation analyses showed that DLGAP1-AS2 expression levels were correlated with tumor differentiation, lymph node metastasis and tumor stage (Fig. 1 F and Tab.S6). Furthermore, univariate and multivariate Cox proportional hazard analyses identified DLGAP1-AS2 as an independent prognostic factor for CRC (Fig. 1G).

To investigate the potential mechanisms mediating DLGAP1-AS2 overexpression in CRC, we analyzed the copy number variations (CNVs) in the TCGA CRC cohort. DLGAP1-AS2 showed an increased genomic copy number in CRC tissues compared with adjacent NCTs, and a weak positive correlation was observed between the expression and copy number of DLGAP1-AS2 in CRC (Fig.S1B). Further experimental validation in 32 paired CRCs and NCTs using qPCR also confirmed the potential regulation of CNV on DLGAP1-AS2 expression (Fig. 1 H-I). In addition, DNA methylation of CpG islands in promoter is an important mechanism regulating gene expression. We analyzed the promoter of DLGAP1-AS2 and failed to observe potential CpG islands, suggesting that the expression of DLGAP1-AS2 was not regulated by DNA methylation (Fig.S1C). Taken together, these data suggest that the aberrant overexpression of DLGAP1-AS2 in CRC is partly regulated by CNV.

### Identification of a novel transcript of DLGAP1-AS2 in CRC

**Fig. 2:**
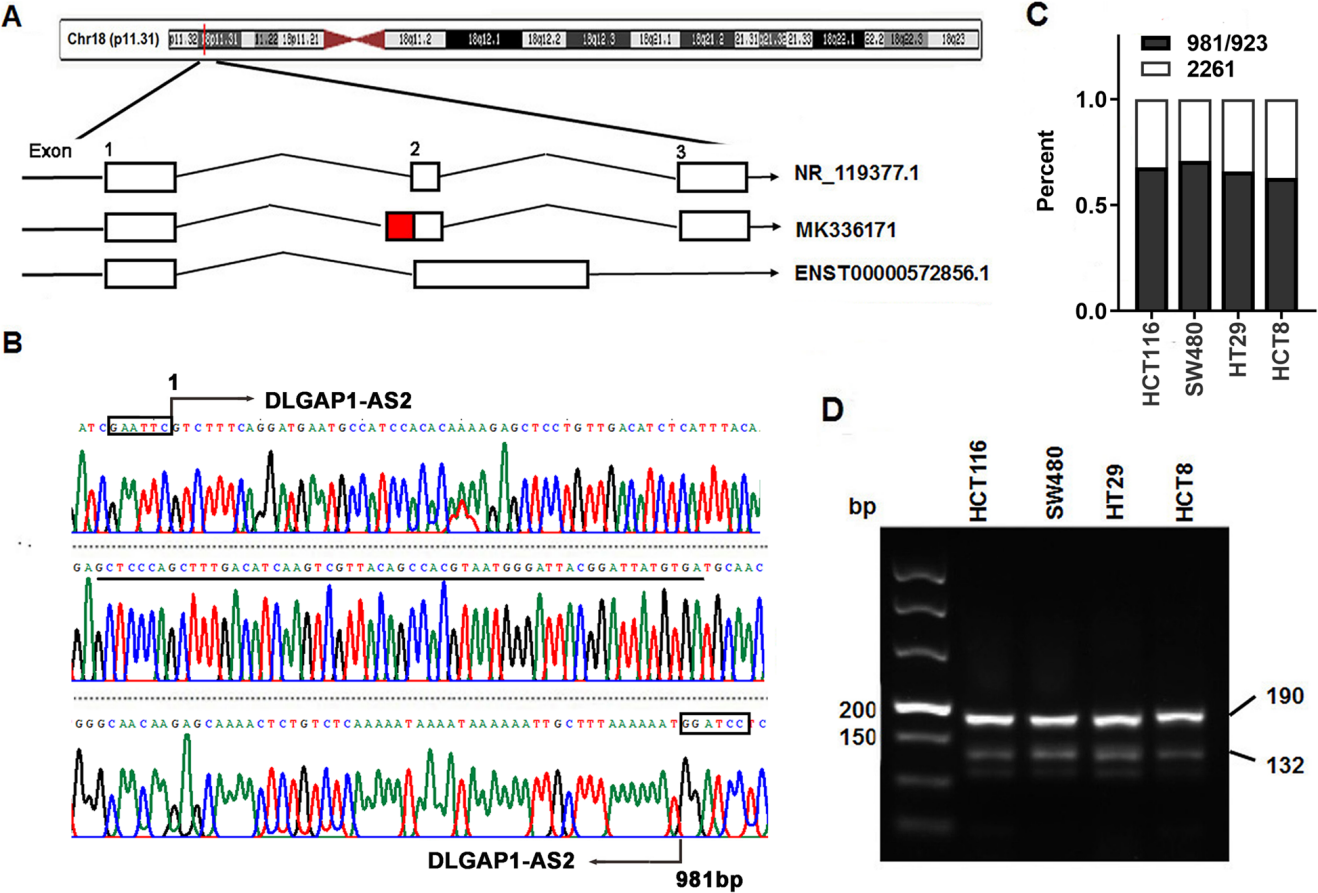
Identification of a novel transcript of DLGAP1-AS2 in CRC. **A** Schematic representation different transcripts of DLGAP1-AS2 and its loci on human chromosome 18p11.31 in the UCSC Genome browser (GRCh38/hg38). **B** The sequencing results of the novel DLGAP1-AS2 transcript. The underlined sequence is the additional 58 bp. **C **The relative abundance of different transcripts of DLGAP1-AS2 in CRC cells. **D** The relative levels of the two transcripts of DLGAP1-AS2 in CRC cell lines were detected with a semi-quantitative RT-PCR method. The bands with 190 bp or 132 bp length represent the 981 bp or 923 bp transcript of DLGAP1-AS2, respectively

Two transcripts with 923 bp (NR_119377.1) or 2261 bp (ENST00000572856.1) in length, seem to be fundamental transcripts of DLGAP1-AS2 based on the comprehensive analyses of DLGAP1-AS2 sequence data from GenBank, GENECODE, and UCSC (Fig. 2 A). Interestingly, when we cloned DLGAP1-AS2 based on the only transcript provided by GenBank (NR_119377.1), a novel transcript with an additional 58 bp in the second exon was identified, which has been submitted to GenBank (MK336171, Fig. 2B). Further analyses using qRT-PCR and semi-quantitative RT-PCR revealed that the novel transcript was the predominant transcript in CRC and other types of cancer cell lines (Fig. 2 C-D and Fig.S1D-E). In addition, further analyses using the Coding Potential Assessment Tool (CPAT) and PhyloCSF indicated that DLGAP1-AS2 lacks protein-coding potential (Fig.S2A-B). Consequently, we focused on this new and predominant transcript for subsequent studies in CRC. DLGAP1-AS2 is highly expressed in different CRC cell lines (Fig.S2C) and distributed in both the cytoplasm and nucleus of CRC cells (Fig.S2D).

### DLGAP1-AS2 promotes CRC growth and metastasis

**Fig. 3:**
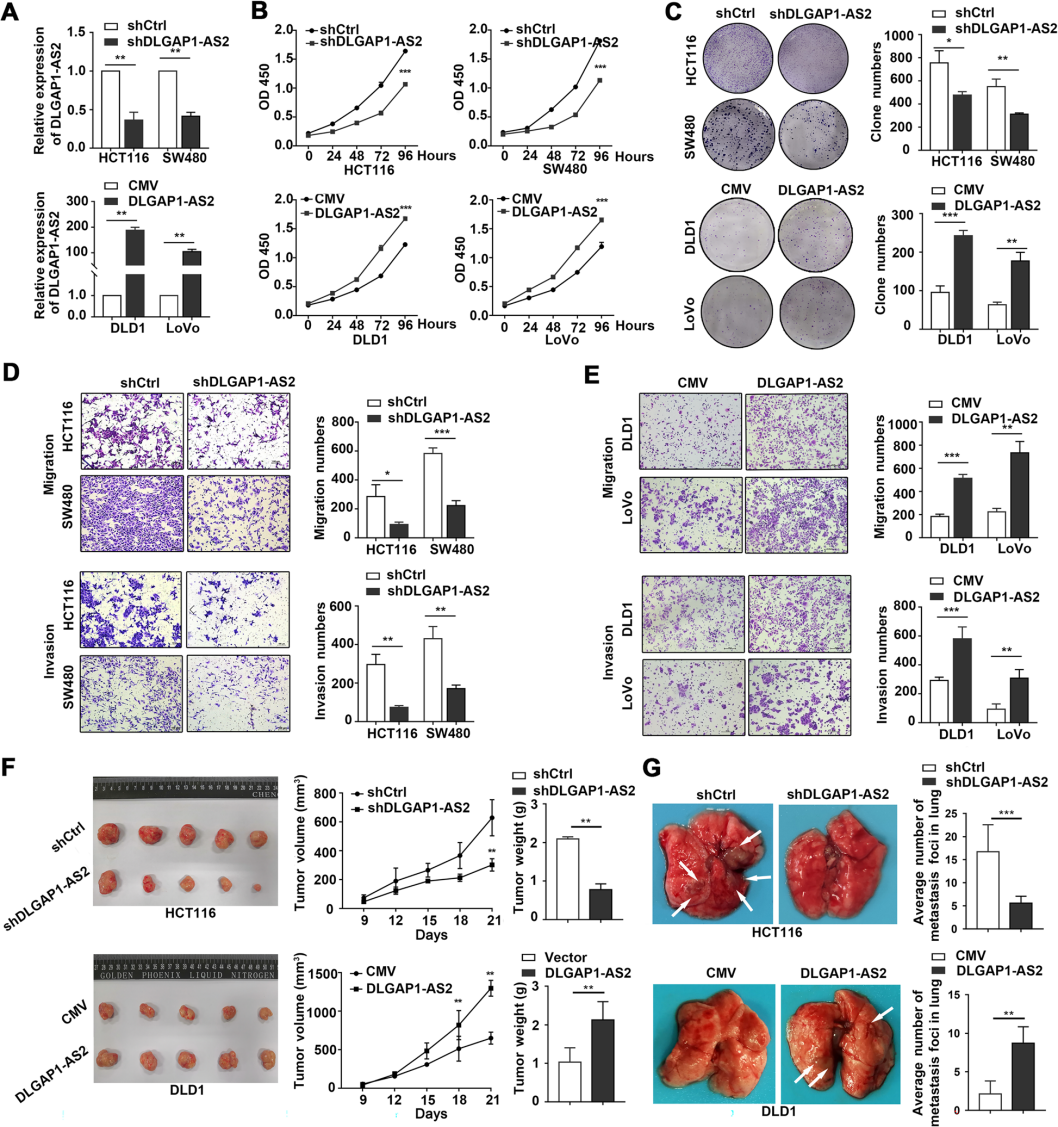
DLGAP1-AS2 promotes CRC proliferation and metastasis. **A** Validations of DLGAP1-AS2 knockdown and overexpression in CRC cells using qRT-PCR. **B-C** The effects of DLGAP1-AS2 on CRC cell proliferation (**B**) and colony formation (**C**) were measured by CCK-8 and colony formation assays, respectively. **D-E** The effects of DLGAP1-AS2 on CRC cell migration and invasion were measured by Transwell assays. **F** The effects of DLGAP1-AS2 on colorectal tumorigenesis were evaluated using the xenograft mouse model (*n* = 5). **G** The effects of DLGAP1-AS2 on CRC metastasis were evaluated in the lung metastasis mouse model (*n* = 5)

CRC cells with relatively higher (HCT116 and SW480) or lower (DLD1 and LoVO) DLGAP1-AS2 expression were selected for gene knockdown or overexpression and subsequent functional assays, respectively. We designed three siRNAs to knockdown DLGAP1-AS2, and the most efficient siRNA sequence (siDLGAP1-AS2-1) was used to construct shRNA vector and cell lines with stable knockdown of DLGAP1-AS2. CCK-8 and colony formation assays demonstrated that DLGAP1-AS2 knockdown significantly inhibited, whereas ectopic DLGAP1-AS2 expression promoted the proliferation and colony formation abilities of CRC cells (Fig. 3 A-C and Fig.S2E-F). Transwell assays showed that DLGAP1-AS2 knockdown drastically inhibited the migration and invasion activities of HCT116 and SW480 cells. In contrast, DLGAP1-AS2 induction enhanced the migration and invasion of DLD1 and LoVo cells (Fig. 3D-E).

To further explore the growth-promoting effects of DLGAP1-AS2 on CRC in vivo, we subcutaneously injected CRC cells with stable knockdown or overexpression of DLGAP1-AS2 into nude mice. Both the volumes and weights of the xenograft tumors in the knockdown group were markedly lower than those in the control group. In contrast, ectopic DLGAP1-AS2 expression significantly promoted CRC tumorigenesis (Fig. 3 F).

We also used a mouse lung metastasis model to evaluate the effect of DLGAP1-AS2 on CRC metastasis. The results showed that DLGAP1-AS2 knockdown drastically inhibited, whereas DLGAP1-AS2 overexpression promoted CRC metastasis (Fig. 3G and Fig.S3A-B). Taken together, these data demonstrate that DLGAP1-AS2 promotes CRC growth and metastasis.

### DLGAP1-AS2 interacts with CPSF2, CSTF3 and ELOA in CRC cells

**Fig. 4:**
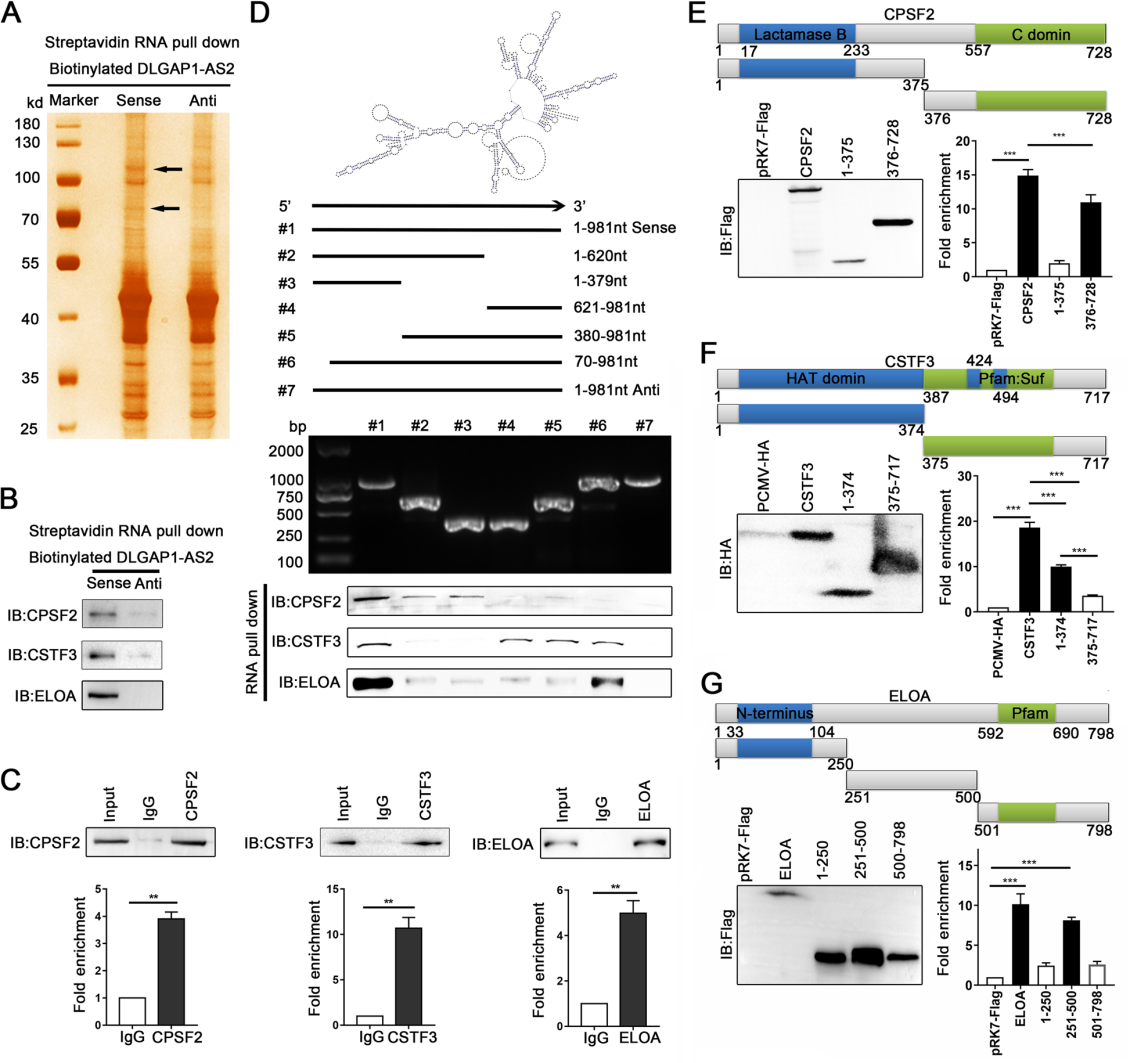
DLGAP1-AS2 physically interacts with CPSF2, CSTF3 and ELOA in CRC cells. **A** Proteins retrieved from the DLGAP1-AS2 RNA pull-down assays were analyzed by SDS–PAGE with silver staining. **B** Immunoblotting analyses of CPSF2, CSTF3 and ELOA in protein retrieved from the DLGAP1-AS2 RNA pull-down assays. **C** RIP assays were performed to confirm the association of CPSF2, CSTF3 and ELOA with DLGAP1-AS2 using the indicated antibodies. qRT-PCR was used to detect the enrichment of DLGAP1-AS2. **D** Immunoblotting of CPSF2, CSTF3 and ELOA in samples from the RNA pull-down assays with full-length or truncated DLGAP1-AS2 RNA probes. **E**-**G** RIP analyses were performed to measure the enrichment of DLGAP1-AS2 by the full-length or truncated proteins of CPSF2 (**E**), CSTF3 (**F**) or ELOA (**G**)

To explore the molecular mechanism underlying the oncogenic role of DLGAP1-AS2 in colorectal carcinogenesis, we performed RNA pull-down assays to identify the proteins associated with DLGAP1-AS2 in CRC cells. The retrieved proteins were subjected to SDS-PAGE electrophoresis, mass spectrum and subsequent western blotting analyses. The results showed that CPSF2, CSTF3 and ELOA were potential DLGAP1-AS2-associated proteins (Fig. 4 A-B and Fig.S4A-C). Moreover, RIP assays further confirmed the associations between these three proteins and DLGAP1-AS2 (Fig. 4 C and Fig.S5).

To identify the regions of DLGAP1-AS2 accounting for the binding to CPSF2, CSTF3 or ELOA, we constructed a series of DLGAP1-AS2 mutants based on their secondary structure as predicted by LNCipedia (http://www.lncipedia.org/) and catRAPID (http://service.tartaglialab.com/page/catrapidgroup) (Fig.S6A-C). RNA pull-down assays with these mutants showed that the 1–379 nt, 621–981 nt or 380–981 nt fragment of DLGAP1-AS2 mediates its interaction with CPSF2, CSTF3 or ELOA, respectively (Fig. 4D).

We then constructed several deletion mutants of these three proteins for RIP assays. The results showed that the deletion of 376–728 aa of CPSF2 significantly abolished the association between CPSF2 and DLGAP1-AS2 (Fig. 4E). Additionally, the 1-374 aa domain of CSTF3 mediates its association with DLGAP1-AS2 (Fig. 4 F), and the 251–500 aa domain of ELOA physically associates with DLGAP1-AS2 in CRC cells (Fig. 4G). Together, these data indicate that DLGAP1-AS2 specifically binds to CPSF2, CSTF3 and ELOA in CRC cells.

### DLGAP1-AS2 promotes ELOA ubiquitination and degradation

**Fig. 5:**
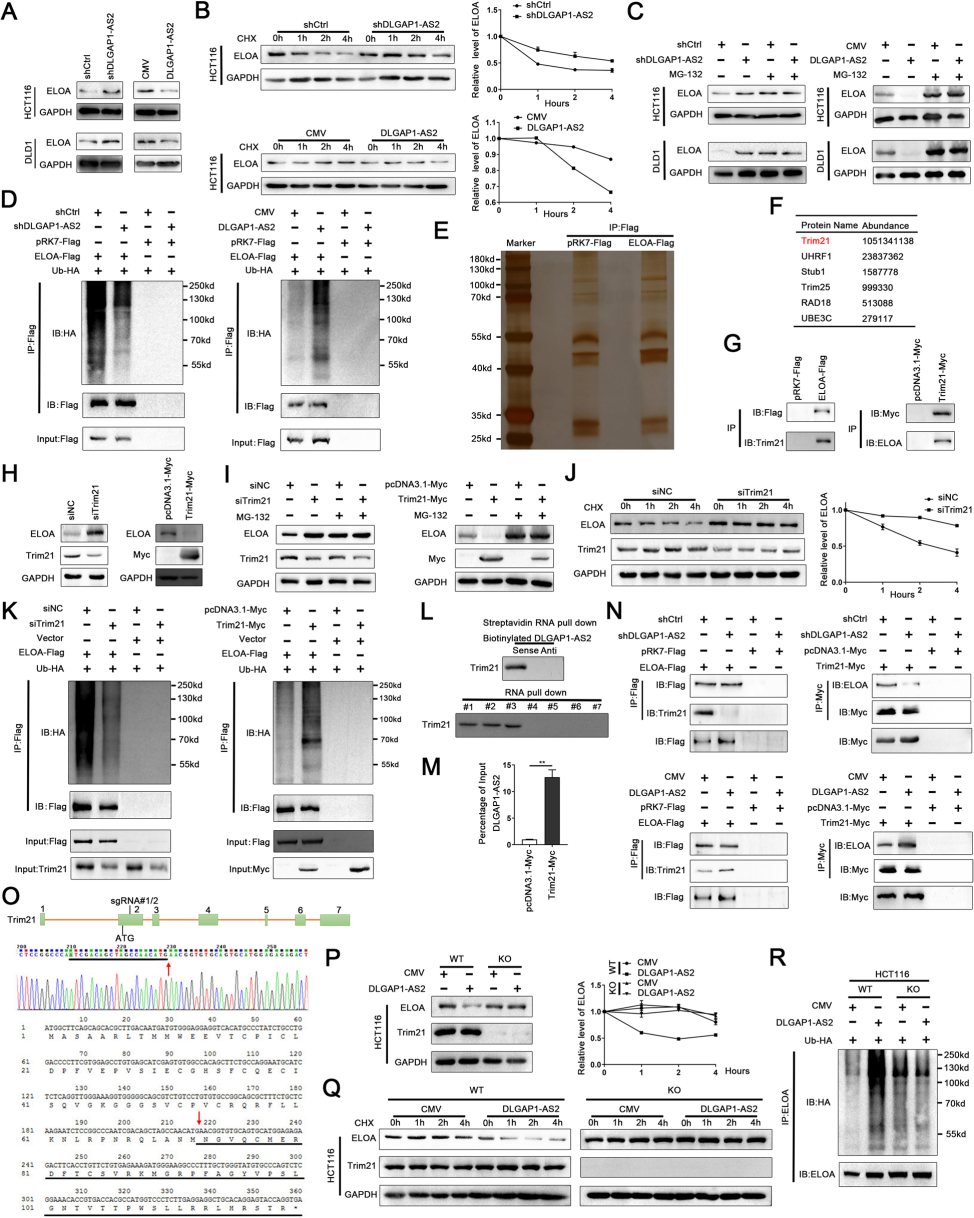
DLGAP1-AS2 promotes Trim21-mediated ELOA ubiquitination and degradation. **A** The effects of DLGAP1-AS2 on the protein levels of ELOA in CRC cells. **B** The effect of knockdown or overexpression DLGAP1-AS2 on ELOA protein stability. Cells were treated with CHX (50 mg/L) for 0, 1, 2 or 4 h before harvest. **C** The protein levels of ELOA were checked in DLGAP1-AS2-depleted or overexpressing CRC cells. Cells were treated with MG132 (20 mmol/L) for 4 h before harvest. **D** DLGAP1-AS2 promoted the ubiquitination modification of ELOA. Cells were co-transfected with ELOA Flag, HA-Ub and/or DLGAP1-AS2 plasmids and treated with MG132 for 4 h before harvest. Ubiquitinated ELOA was measured by western blotting using an anti-HA antibody following the immunoprecipitation of Flag. **E** Proteins retrieved from the ELOA-flag immune coprecipitation assays were analyzed by SDS–PAGE with silver staining. **F** E3 ligases screened by the mass spectrometry with immunoprecipitation of ELOA. **G** Interactions between ELOA and Trim21 were verified by immunoprecipitation in HCT116 cells. **H** The effects of Trim21 knockdown or overexpression on the protein levels of ELOA in HCT116 cells. **I** MG132 treatment rescued Trim21-induced downregulation of ELOA. **J** Trim21 knockdown increased the half-life of ELOA in HCT116 cells treated with CHX. **K** Ectopic Trim21 expression promoted the ubiquitous modification of ELOA. **L** Immunoblotting analyses of Trim21 in protein retrieved from the DLGAP1-AS2 RNA pull-down assays. The detailed information of DLGAP1-AS2 RNA probes (#1-#7) was shown in Fig. 4D. **M** RIP assays were performed to confirm the association of Trim21 with DLGAP1-AS2. **N** DLGAP1-AS2 enhanced the interactions between ELOA and Trim21. **O** Schematic representation of sgRNA-CRISPR/Cas9 target sites in TRIM21 and preterminal sites in the stable Trim21-KO HCT116 cells. **P** The effect of DLGAP1-AS2 on the protein levels of ELOA in Trim21-KO HCT116 cells. **Q** The effect of DLGAP1-AS2 on the half-life of ELOA in Trim21-KO HCT116 cells. **R** The effect of DLGAP1-AS2 on the ubiquitination modification of ELOA in Trim21-KO HCT116 cells

Although we revealed that DLGAP1-AS2 interacted with CPSF2, CSTF3 and ELOA in CRC cells, their underlying functional and mechanistic effects were unclear. We evaluated the effects of DLGAP1-AS2 on the expression of these targets, and no obvious changes were observed at either the protein or mRNA levels of CPSF2 and CSTF3. The mRNA levels of ELOA also did not change in DLGAP1-AS2-depleted or DLGAP1-AS2-overexpressing CRC cells (Fig.S7A-D). However, the protein levels of ELOA were dramatically increased in DLGAP1-AS2-depleted CRC cells and were notably reduced with DLGAP1-AS2 overexpression (Fig. 5 A). Moreover, DLGAP1-AS2 depletion increased, whereas ectopic DLGAP1-AS2 expression decreased the half-life of ELOA protein in CRC cells treated with the protein synthesis inhibitor cycloheximide (CHX) (Fig. 5B and Fig.S8A). DLGAP1-AS2-induced downregulation of ELOA protein was blocked in CRC cells treated with the proteasome inhibitor MG132 (Fig. 5 C). Furthermore, we found that the ubiquitination levels of both exogenous and endogenous ELOA were significantly increased in DLGAP1-AS2 overexpressing cells and were significantly decreased in DLGAP1-AS2 depleted cells (Fig. 5D and Fig.S8B). Taken together, these data suggest that DLGAP1-AS2 promotes the proteasome-dependent degradation of ELOA in CRC cells.

### DLGAP1-AS2 promotes the interactions between ELOA and Trim21

To investigate how DLGAP1-AS2 accelerates the ubiquitin-mediated proteasome degradation of ELOA, we screened out six E3 ligases using ELOA-Flag IP and subsequent mass spectrometry analyses (Fig. 5E-F). Of these E3 ligases, Trim21 showed the highest abundance, and co-IP assays further confirmed the association between Trim21 and ELOA (Fig. 5G). In addition, both ELOA and Trim21 localized in the cytoplasm of CRC cells, whereas CPSF2 and CSTF3 were mainly located in the nucleus, suggesting that there was no obvious competitive binding to DLGAP1-AS2 for these proteins (Fig.S8C). The protein levels of ELOA were dramatically increased in Trim21-depleted CRC cells and were significantly reduced in Trim21-overexpressing CRC cells (Fig. 5 H). MG132 treatment rescued the Trim21-induced downregulation of ELOA (Fig. 5I), suggesting that Trim21 promotes the proteasome-dependent degradation of ELOA in CRC cells. Moreover, Trim21 knockdown increased the half-life of ELOA in CRC cells treated with CHX (Fig. 5 J). Furthermore, we demonstrated that the ubiquitination levels of ELOA were significantly decreased in Trim21-depleted CRC cells and increased in Trim21-overexpressing CRC cells (Fig. 5 K).

RNA pull-down assays revealed that the 1–379 nt fragment of DLGAP1-AS2 mediates the bind of DLGAP1-AS2 to Trim21 (Figs. 4D and 5 L), and RIP assays further indicated that DLGAP1-AS2 was significantly enriched in the RNA-protein complexes precipitated with anti-Myc antibody in CRC cells (Fig. 5 M). We next examined whether DLGAP1-AS2 impacts the interactions between ELOA and Trim21 in CRC cells, and showed that DLGAP1-AS2 knockdown significantly impaired, whereas ectopic DLGAP1-AS2 expression significantly enhanced this association (Fig. 5 N). Collectively, these results indicate that DLGAP1-AS2 promotes Trim21-mediated ubiquitination degradation of ELOA by enhancing the interaction between Trim21 and ELOA.

We established a Trim21-KO HCT116 cell line via CRISPR/Cas9 system, which contained a 52 bp deletion that also led to a frame shift mutation of TRIM21 (Fig. 5O). In Trim21-KO cells, manipulating DLGAP1-AS2 failed to affect the expression, half-life and ubiquitinationof ELOA, confirming that DLGAP1-AS2 regulates the ubiquitination degradation of ELOA through Trim21 (Fig. 5P-R).

### ELOA inhibits CRC growth and metastasis

**Fig. 6:**
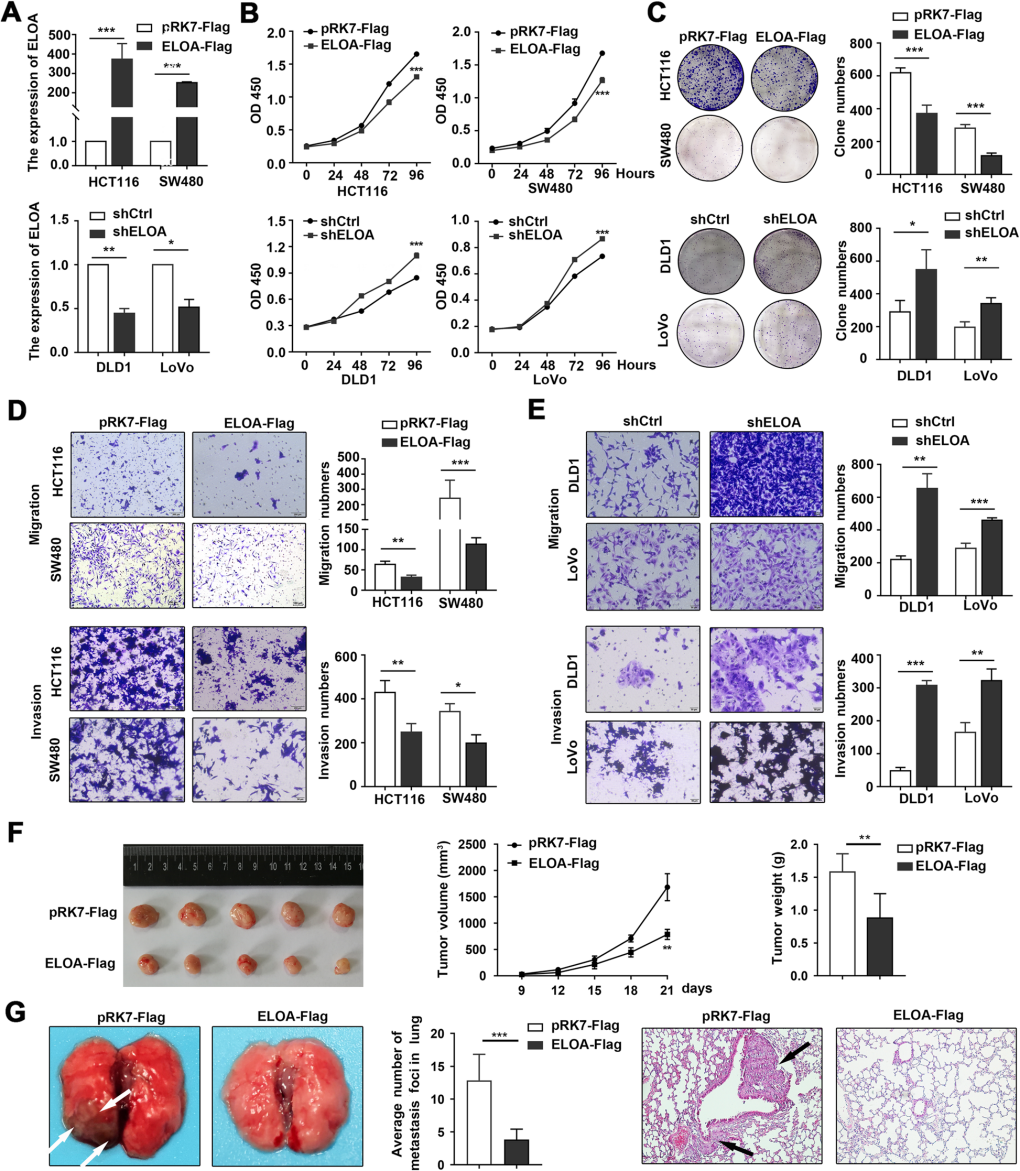
ELOA inhibits CRC proliferation and metastasis. **A** Validations of ELOA overexpression and knockdown in CRC cells using qRT-PCR. **B-C** The effects of ELOA on cell proliferation (**B**) and colony formation(**C**). **D-E** The effects of ELOA on CRC cell migration and invasion were measured by Transwell assays. **F** The effects of ELOA overexpression on CRC growth were evaluated using a xenograft mouse models (*n* = 5). **G** The effect of ELOA on CRC metastasis was evaluated using a lung metastasis mouse model (*n* = 5)

ELOA, a subunit of the transcription factor B (SIII) complex, is relatively understudied and its role in tumorigenesis and progression is unclear. Therefore, we studied the functional role of ELOA in CRC by using a series of in vitro and in vivo assays. We demonstrated that ectopic ELOA expression inhibited, whereas ELOA knockdown significantly promoted CRC cell proliferation and colony formation (Fig. 6 A-C and Fig.S9A-B). Transwell assays demonstrated the inhibitory functions of ELOA on the migratory and invasive abilities of CRC cells (Fig. 6D-E).

We further confirmed the growth-suppressive effects of ELOA on CRC in vivo by using a xenograft nude mouse model (Fig. 6 F). In addition, a mouse lung metastasis model was applied to evaluate the effect of ELOA on CRC metastasis. The results showed that the number of lung metastatic nodules was decreased in the ELOA-overexpressing group compared with the control group (Fig. 6G). Collectively, the above results reveal that ELOA inhibits the growth and metastasis of CRC.

### ELOA protein expression negatively correlates with DLGAP1-AS2 and is associated with good prognosis in CRC

**Fig. 7:**
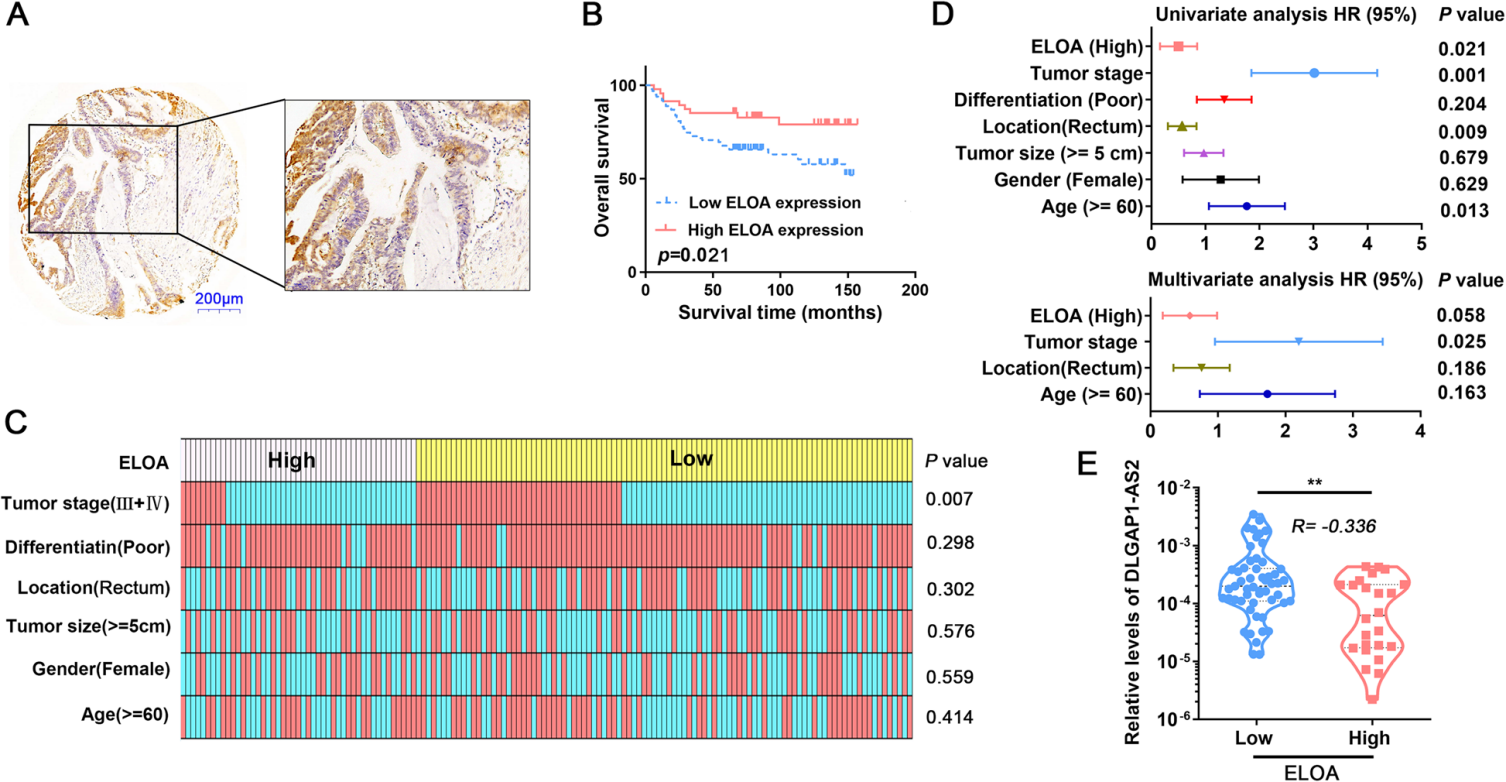
ELOA is associated with good clinical outcomes in CRC patients. **A** ELOA protein expression in CRC tissues was detected by IHC. **B** Kaplan–Meier survival analyses of overall survival according to the ELOA protein levels in CRC tissues. **C** The relationship between ELOA expression and clinicopathological parameters in CRC. **D** Univariate and multivariate regression analyses in CRC patients. **E** The relationship between DLGAP1-AS2 expression and ELOA protein expression in CRC tissues

To further evaluate the role of ELOA in CRC, we detected its expression in clinical CRC tissues using IHC (Fig. 7 A). Kaplan-Meier survival analyses showed that low ELOA expression was significantly correlated with poor overall survival (Fig. 7B). Correlation analyses showed that ELOA expression levels were correlated with tumor stage (Fig. 7 C and Tab.S7). Furthermore, univariate and multivariate Cox proportional hazard analyses identified ELOA as a potential prognostic factor for CRC (Fig. 7D). Importantly, a significant negative correlation was observed between the expression levels of ELOA protein and DLGAP1-AS2 (Fig. 7E). These results suggest that ELOA is involved in CRC tumorigenesis.

### ELOA is a downstream functional target of DLGAP1-AS2

**Fig. 8:**
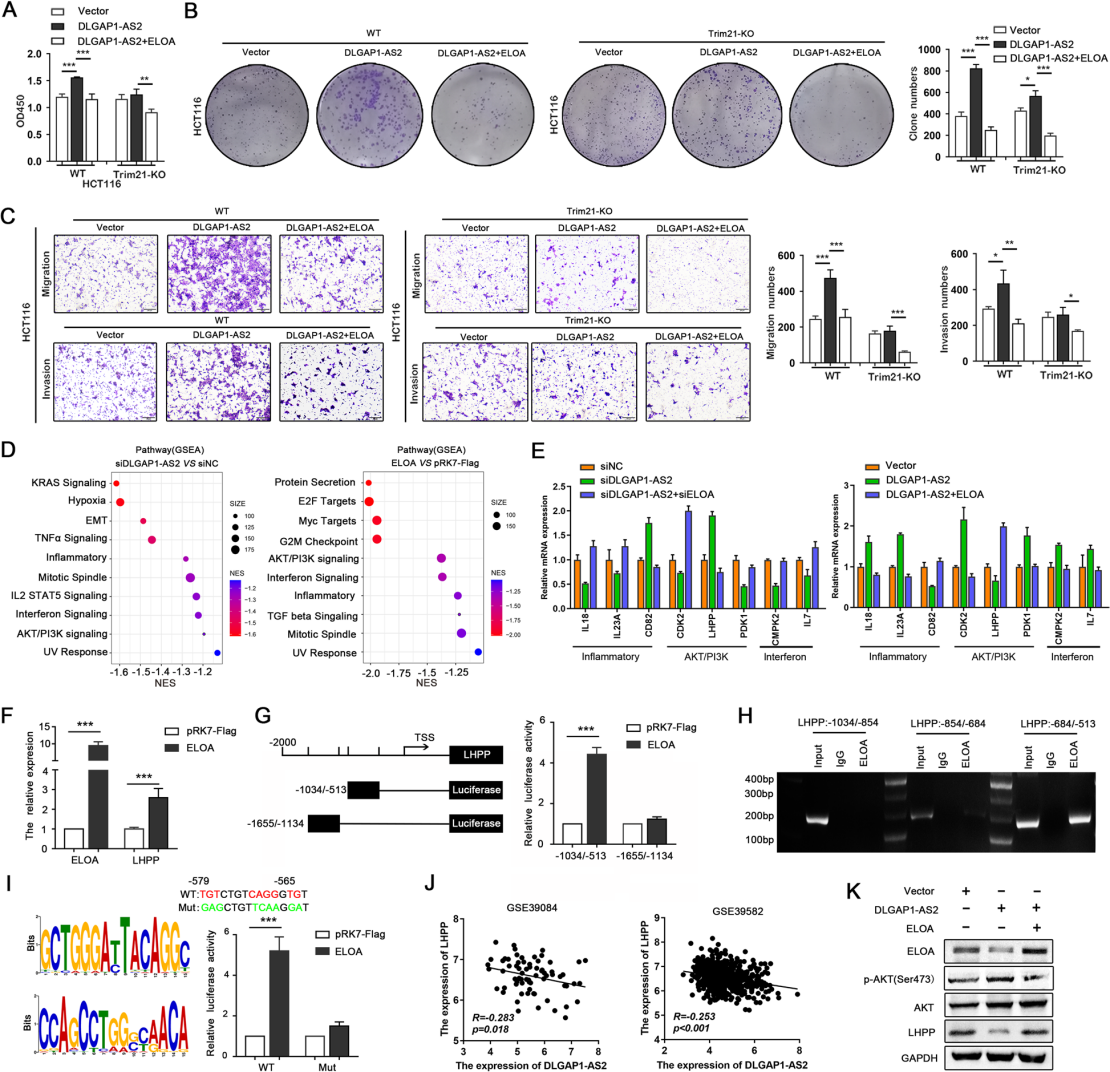
ELOA mediates DLGAP1-AS2-driven CRC growth and metastasis. **A-B** Cell proliferation and colony formation abilities in WT and Trim21-KO HCT116 cells transfected with plasmids of DLGAP1-AS2 and ELOA. **C** Cell migration and invasion abilities in WT and Trim21-KO HCT116 cells transfected with plasmids of DLGAP1-AS2 and ELOA. **D** GSEA pathway enrichment analyses using the differentially expressed genes (DRGs) potentially affected by DLGAP1-AS2 and ELOA. DRGs were identified in DLGAP1-AS2-silenced and ELOA-overexpressing HCT116 cells using RNA-seq. **E** Validation of selected DRGs in HCT116 cells transfected with the siRNAs or plasmids of DLGAP1-AS2 and ELOA. **F** LHPP mRNA expression was detected by qRT-PCR in ELOA-overexpressing HCT116 cells. **G** The relative luciferase activity of LHPP promoter in ELOA-overexpressing HCT116 cells. **H** The recruitment of ELOA to the indicated regions of the LHPP promoter was determined by ChIP-PCR. IgG was used as the negative control. **I** The potential binding motif of ELOA was validated by luciferase assays. Mut: the mutant LHPP promoter lacking of the putative binding motif of ELOA. **J** Correlation analyses between DLGAP1-AS2 and LHPP. **K** The effect of DLGAP1-AS2/ELOA on the AKT pathway (pAKT at Ser473)

To verify whether ELOA is a functional target of DLGAP1-AS2, we performed a rescue assay. The results revealed that silencing ELOA expression restored the impaired proliferation and colony formation abilities induced by DLGAP1-AS2 knockdown, whereas ectopic expression of ELOA remarkably impaired the proliferation-promoting effects of DLGAP1-AS2 overexpression in HCT116 cells (Fig.S9C-D). Transwell assays demonstrated that ELOA block the promoting effect of DLGAP1-AS2 on the migration abilities of CRC cells (Fig.S9E). What is more, the tumor-promoting effects of DLGAP1-AS2 by inhibiting ELOA were observed in WT but not in Trim21-KO CRC cells (Fig. 8 A-C). These data confirm that DLGAP1-AS2 exerts cancer-promoting effects by regulating the Trim21-ELOA axis in CRC.

To further investigate the mechanistic association between DLGAP1-AS2 and ELOA, we compared the transcriptome profiles in HCT116 cells transfected with si-DLGAP1-AS2, ELOA plasmid or their corresponding control. A total of 682 DEGs, including 478 upregulated genes and 204 downregulated genes, were identified in DLGAP1-AS2-silenced HCT116 cells compared with control cells. In contrast, a total of 1420 DEGs, including 768 downregulated genes and 652 upregulated genes, were observed in ELOA-overexpressing HCT116 cells. We performed GSEA pathway enrichment analyses using these DEGs and found that the pathways potentially affected by DLGAP1-AS2 or ELOA highly overlapped, including the inflammatory response, mitotic spindle, PI3K/AKT, interferon and UV response pathways (Fig. 8D). Furthermore, some genes in these pathways potentially regulated by both DLGAP1-AS2 and ELOA were verified using qRT-PCR. We observed that ELOA overexpression significantly rescued the effects of DLGAP1-AS2 knockdown on the expression of several genes in the inflammatory response, AKT and interferon pathways (Fig. 8E), suggesting that DLGAP1-AS2 regulates the expression of these genes through ELOA.

### ELOA transcriptionally regulates LHPP expression by specifically binding to its promoter

To screen gene harboring the specific binding site of ELOA in their promoters, a ChIP-on-chip assay was employed in HCT116 cells using Nimblegen human 720 K RefSeq promoter arrays. A total of 836 promoters were enriched by the ChIP-on-chip assay using an ELOA antibody (false discovery rate < 0.05). By combining the results of the ChIP-on-chip assay with those of the above-mentioned transcriptome profile data, three genes (LHPP, IL7 and CMPK2) potentially regulated by ELOA were screened out. Of them, only LHPP, a newly discovered tumor suppressor [16], appeared to be upregulated by ELOA and was selected for further validations. As expected, LHPP mRNA expression was significantly increased in ELOA-overexpressing CRC cells (Fig. 8 F).

To validate the potential ELOA-binding region (-1034/-513) revealed by the ChIP-on-chip assay, we constructed two mutants with  -1034/-513 or -1655/-1134 (as a negative control) of the LHPP promoter. Luciferase assays showed that ELOA stimulated the reporter expression of the constructs containing the  -1034/-513 of the LHPP promoter, suggesting that ELOA transcriptionally regulated LHPP expression by specifically binding to its promoter (Fig. 8G). To validate it, we performed ChIP-qPCR assays using primers flanking the promoter segments of LHPP (-1034/-513). We observed a significant amount of ELOA bound to the  -684/-513 region of the LHPP promoter (Fig. 8 H), confirming that ELOA enhances the transcription of LHPP by directly binding to its promoter.

Using MEME-ChIP database and Markov model, we searched the conserved sequences in the promoters of 836 genes screened by the ChIP-on-chip assay, and revealed two possible motif binding sequences of ELOA (GCTGGGATTACAGGC and CCAGCCTGGGCAACA). One of them existed in the  -579/-565 region of the LHPP promoter (TGTCTGTCAGGGTGT, partially reverse complement to the sequence of CCAGCCTGGGCAACA). When this region was mutated, ELOA failed to induce luciferase expression of the recombinant reporter plasmids (Fig. 8I). Based on the above results, we conclude that ELOA promotes the transcription of LHPP by binding to the  -579/-565 region of the LHPP promoter.

### DLGAP1-AS2 activates the AKT pathway by regulating the ELOA/LHPP axis

We revealed that DLGAP1-AS1 regulated several key cancer-related pathways, including PI3K/AKT. Interestingly, recent studies have reported that LHPP inhibits the PI3K/AKT signaling pathway in CRC [17, 18]. We found that LHPP was expressed at lower levels in CRC tissues compared with NCTs based on multiple public CRC cohorts (Fig.S10A). We also showed that LHPP expression was negatively correlated with DLGAP1-AS2, and was positively correlated with ELOA in CRC (Fig. 8 J and Fig.S10B). To determine whether LHPP mediated the regulation of the PI3K/AKT pathway by ELOA in CRC, we measured the AKT activity (pAKT at Ser473 and Thr308) in ELOA-overexpressing CRC cells and confirmed that ELOA increased LHPP expression and suppressed AKT pathway activity (Fig.S10C). What is more, the rescue assays revealed that ELOA overexpression inhibited DLGAP1-AS2-induced AKT activation (pAKT at Ser473 but not at Thr308) (Fig. 8 K and Fig.S10D). Taken together, these data demonstrate that DLGAP1-AS2 promotes CRC development and progression by regulating the ELOA/LHPP/AKT signaling axis in CRC.

### CPSF2 and CSTF3 bind to DLGAP1-AS2 and increase its stability

**Fig. 9:**
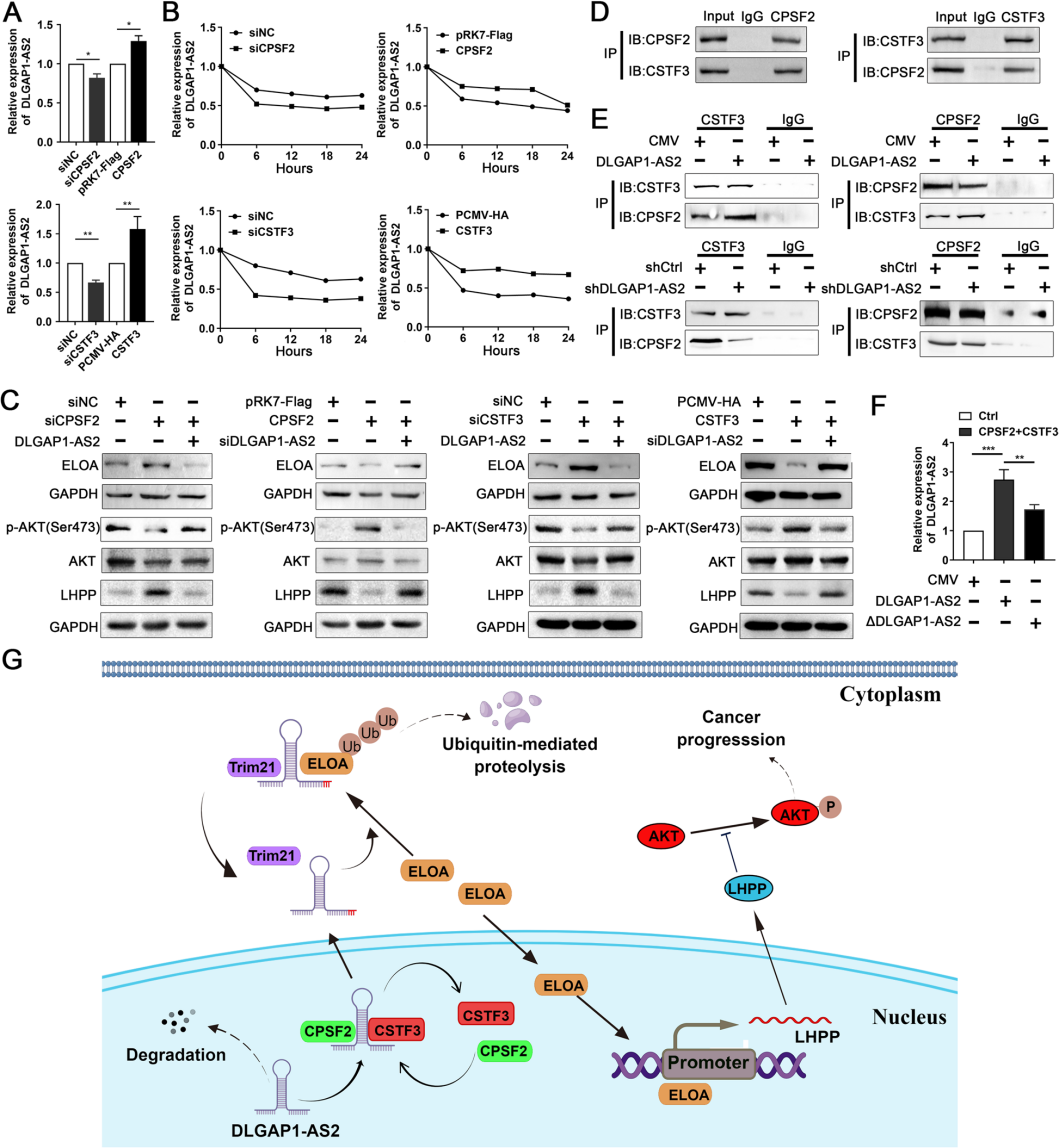
CPSF2 and CSTF3 increase the stability of DLGAP1-AS2 in CRC cells. **A** The effects of CPSF2 or CSTF3 on DLGAP1-AS2 expression. **B** The effect of CPSF2 or CSTF3 on the half-life of DLGAP1-AS2 in CRC cells treated with actinomycin D (2ug/ml). **C** The effect of CPSF2 or CSTF3 on the expression of ELOA and AKT pathway (pAKT at Ser473). **D** The interaction between CPSF2 and CSTF3 was evaluated using IP assays. **E** DLGAP1-AS2 promoted the interaction between CPSF2 and CSTF3. **F** The effect of CPSF2 and CSTF3 overexpression on the expression of DLGAP1-AS2. DLGAP1-AS2 mutation (ΔAATAAA) inhibited CPSF2 and CSTF3-induced upregulation of DLGAP1-AS2 levels in HCT116 cells. **G** Integrated model depicting the tumor-promoting effect and molecular mechanism of DLGAP1-AS2 in CRC

CPSF2 and CSTF3, which are part of the C/P machinery family, function as multi protein complexes to regulate RNA processing and stability. We have shown that both CPSF2 and CSTF3 are DLGAP1-AS2-associated proteins. Analyses of public CRC databases revealed that both CPSF2 and CSTF3 were upregulated in tumor tissues compared with paired NCTs, and higher CPSF2 and CSTF3 expression was associated with worse survival (Fig.S11A-D). We observed that DLGAP1-AS2 did not affect the expression of CPSF2 and CSTF3 in CRC cells (Fig.S7A-D), whereas CPSF2 or CSTF3 positively regulated the expression of DLGAP1-AS2 in CRC cells (Fig. 9 A). Moreover, actinomycin D (2ug/ml) treatment assays showed that both CPSF2 and CSTF3 increased the stability of DLGAP1-AS2 (Fig. 9B). As expected, CPSF2 or CSTF3 also negatively regulated the expression of ELOA and activated AKT signaling (pAKT at Ser473 but not at Thr308) through DLGAP1-AS2 in CRC cells (Fig. 9 C and Fig.S12A-C).

We further revealed that CPSF2 was able to bind to CSTF3 (Fig. 9D), which was enhanced by DLGAP1-AS2 in CRC cells (Fig. 9E), suggesting that DLGAP1-AS2 functions as a molecular scaffold to enhance the association between them. In addition, we showed that CPSF2 and CSTF3 might work together to increase the levels of DLGAP1-AS2. Interestingly, we found a CPSF2-specific binding motif (AAUAAA) in the 89–94 nt region of DLGAP1-AS2. When the motif was deleted (ΔDLGAP1-AS2), the promoting effect of CPSF2 and CSTF3 on DLGAP1-AS2 was significantly suppressed (Fig. 9 F). Taken together, these data suggest that CPSF2 and CSTF3 may work together to stabilize DLGAP1-AS2 in CRC cells.

## Discussion

The identified number of lncRNA increased rapidly with the progress of RNA sequencing technology. In our previous studies, we screened and identified several tumor-related lncRNAs in CRC using microarrays [7–15]. In this study, we further screened CRC-related lncRNAs using next-generation sequencing and identified a cancer-promoting lncRNA, DLGAP1-AS2, is upregulated in many types of cancers.

DLGAP1-AS2 is located at chromosome 18p11.31, and a total of three transcripts of DLGAP1-AS2 have been included in GENECODE or GenBank. In this study, we identified a novel DLGAP1-AS2 transcript that was the predominant transcript in CRC and other types of cancer cells. Although the expression levels of the other two transcripts of DLGAP1-AS2 ((NR_119377.1 and ENST00000572856.1) were much lower than that of the identified novel transcript in most cancer cells checked, our preliminary functional investigations showed that they also showed cancer-promoting functions in CRC cells (data not shown), suggesting that they may also play a role under specific psychological and pathological conditions.

Based on multiple CRC cohorts, we showed that DLGAP1-AS2 is a promising prognostic biomarker for CRC. Functionally, we demonstrated that DLGAP1-AS2 has strong oncogenic activity by promoting CRC tumorigenicity and progression. Recent studies also reported the upregulation and prognostic value of DLGAP1-AS2 in several cancer types. Similar tumor-promoting functions have also been observed in multiple cancer types, including rectal cancer [19–26]. For example, a recent study conducted by Zeng et al. reported that DLGAP1-AS2 promotes the radioresistance in rectal cancer stem cells through regulating the E2F1-CD151 axis [27]. These findings indicate that DLGAP1-AS2 is a potential oncogene, and may act as a pancancer therapeutic target and biomarker. However, the detailed functions and mechanisms of DLGAP1-AS2 in CRC were still unclear.

LncRNAs typically exert their biological functions through physical interactions with DNA, RNA, or proteins [28]. Little was known about the molecular mechanisms of DLGAP1-AS2 in cancers. Limited studies reported that DLGAP1-AS2 sponges and inhibits the activities of miR-503 or miR-505. In addition, a recent study reported that DLGAP1-AS2 binds to the transcription factor Six3 and inhibits its occupancy in Wnt1 promoter, resulting in the activation of Wnt/β-catenin signaling in gastric cancer cells. In view of relative low abundance of miR-503 and miR-505 and mainly cytoplasm location of DLGAP1-AS2 in CRC cells, we speculated that there are other mechanisms mediating the cancer-promoting functions of DLGAP1-AS2 in CRC.

In this study, we identified CPSF2, CSTF3 and ELOA as bona fide interacting partners of DLGAP1-AS2. ELOA was originally identified as a transcriptionally active subunit of RNA polymerase II (Pol II) transcription factor Elongin (SIII), which stimulates the overall rate of Pol II elongation through direct interactions with the enzyme [29, 30]. Elongin is composed of ELOA and a heterodimeric submodule comprised of Elongin B and C proteins, which bind to a short ELOA sequence motif referred to as the BC box that has potent transcriptional regulation activity [31]. In addition to being a part of SIII, ELOA also functions as the substrate recognition subunit of a Cullin-RING E3 ubiquitin ligase that ubiquitinates the RPB1 subunit of Pol II in response to DNA damage.

Although Elongins B and C have been well characterized in tumorigenesis, little is known about the role of ELOA in tumorigenesis and how its activity is regulated. By a series of experimental validations, we identified ELOA as a key downstream target of DLGAP1-AS2.Here, we revealed that DLGAP1-AS2 enhances ELOA ubiquitination and degradation by promoting the binding of the E3 ligase Trim21 to ELOA, revealing a novel regulatory mechanism for ELOA at the posttranslational level. Using a series of in vitro and in vivo assays, we revealed, for the first time, the inhibitory functions of ELOA in CRC growth and metastasis, discovering a novel role of ELOA in tumorigenesis and progression.

By expression profile analyses, we demonstrated that DLGAP1-AS2 and ELOA share highly similar regulatory effects on the patterns of signaling pathways. Moreover, rescue assays also confirmed that ELOA is a downstream target of DLGAP1-AS2. As a family of transcription elongation factors, ELOA has been shown to play a critical role in optimal gene induction under stress conditions or developmental stimuli [32]. Little is known about the downstream targets of ELOA in cancer cells. We reveled ELOA as a novel transcription regulator for LHPP, a newly identified tumor suppressor [16]. LHPP impedes tumor proliferation and metastasis and inhibits cancer progression through the PI3K/AKT pathway by regulating the phosphorylated AKT at Ser473 in multiple cancer types [33–38]. In this study, for the first time, we provided evidence that ELOA directly binds to the specific promoter region of LHPP and promotes its transcription, resulting in increased LHPP expression and decreased AKT activity.

Although several studies have reported aberrant upregulation of DLAGP1-AS2 in human cancers, its underlying mechanism is unclear. CPSF2 and CSTF3 reported to be members of the cytoplasmic polyadenylation element(CPF) family, which are parts of a multi protein complex essential for the formation of the mRNA 3’ end-processing machinery [39]. CPSF2 is the 100 kDa subunit of CPSF, which consists of four polypeptides. CPSF2 recognizes the AAUAAA polyadenylation signal, which determines the site of 3’-end cleavage interactions within pre mRNAs and plays a key role in nuclear export, translational initiation and transcript stability [40]. Whereas CSTF3 binds GU-rich sequences located downstream from the cleavage site AAUAAA and adds a poly(A) tail for polyadenylation, resulting in increased RNA stability [41]. Here, we proved that CPSF2 and CSTF3 bind to DLGAP1-AS2 and enhance its stability, thereby inhibiting the ELOA/LHPP axis in CRC. Our data reveal a posttranscriptional regulatory mechanism that, at least partially, mediates the upregulation of DLGAP1-AS2 in CRC. However, how CPSF2 and CSTF3 regulate the stability of DLGAP1-AS2 is not clear, and whether the regulatory mechanism could be validated in other cancer types also remains to be elucidated.

## Conclusion

In conclusion, we demonstrated that DLGAP1-AS2 functions as an oncogenic lncRNA in CRC by promoting tumor growth and metastasis. Mechanistically, DLGAP1-AS2 inhibits ELOA protein stability by promoting Trim21-mediated ubiquitination and degradation of ELOA. We also demonstrated that ELOA enhances the transcription of LHPP by binding to its promoter and thereby revealed uncovered a novel regulatory axis of DLGAP1-AS2/Trim21/ELOA/LHPP in CRC. In addition, we uncovered that CPSF2 and CSTF3 bind to DLGAP1-AS2 and increase its stability. These data provide insight into colorectal carcinogenesis and suggest DLGAP1-AS2 as a promising prognostic biomarker and therapeutic target for CRC (Fig. 9G).

## Supplementary Information

**Additional file 1: Fig. S1.** The expression and transcript analyses of DLGAP1-AS2. **Fig. S2.** Characterization of the protein-coding potential, subcellular localization and function for DLGAP1-AS2 in CRC. **Fig. S3.** HE staining for lung tissues from a lung metastasis mouse model. **Fig. S4.** Mass spectrometry analyses in samples from streptavidin pull down assays. **Fig. S5.** RIP assays. **Fig. S6.** Graphic illustration of predicted DLGAP1-AS2 secondary structure. **Fig. S7.** The effect of DLGAP1 on CPSF2, CSTF3 and ELOA in CRC. **Fig. S8.** DLGAP1-AS2 promoted the degradation of ELOA. **Fig. S9.** The function of ELOA andELOA as the downstream of DLGAP1-AS2. **Fig. S10.** The expression of LHPP and its relationship to ELOA in CRC. **Fig. S11.** CPSF2 and CSTF3 are associated with poor clinical outcomes in patients with CRC. **Fig. S12.** The effect of CPSF2 or CSTF3 on AKT. **Tab. S1.** Primer sequence. **Tab. S2.** siRNA sequences. **Tab. S3.** Relatedsequence information for plasmid construction. **Tab. S4.** The sequences for DLAGP1-AS2 and its deletion fragments used in vitro transcription. **Tab. S5.** Antibody information. **Tab. S6.** Relationships between DLGAP1-AS2 expression and clinical pathologic factors of the patients with CRC. **Tab. S7.** Relationships between ELOA expression and clinical pathologic factors of the patients with CRC.

## Data Availability

The datasets used and/or analyzed during the current study are available from the corresponding author on reasonable request.

## Abbreviations

ChIP: Chromatin immunoprecipitation
CPSF2: Cleavage and polyadenylation specificity factor
CRC: Colorectal cancer
CSTF3: Cleavage stimulation factor
ELOA: Elongin A
IHC: Immunohistochemistry
LHPP: Phospholysine phosphohistidine inorganic pyrophosphate phosphatase
LncRNAs: Long noncoding RNAs
qRT-PCR: Quantitative realtime polymerase chain reaction
Trim21: Tripartite motif containing 21
RIP: RNA immunoprecipitation
TCGA: The Cancer Genome Atlas
