# Supplementary material for: Long noncoding RNA DLGAP1-AS2 promotes tumorigenesis and metastasis by regulating the Trim21/ELOA/LHPP axis in colorectal cancer
Source: Mol Cancer. 2022 Nov 14;21:210. doi: 10.1186/s12943-022-01675-w (PMC9664729; doi:10.1186/s12943-022-01675-w)
Supplement: Supplementary file 1 — Additional file 1: Fig. S1. The expression and transcript analyses of DLGAP1-AS2. Fig. S2. Characterization of the protein-coding potential, subcellular localization and function for DLGAP1-AS2 in CRC. Fig. S3. HE staining for lung tissues from a lung metastasis mouse model. Fig. S4. Mass spectrometry analyses in samples from streptavidin pull down assays. Fig. S5. RIP assays. Fig. S6. Graphic illustration of predicted DLGAP1-AS2 secondary structure. Fig. S7. The effect of DLGAP1 on CPSF2, CSTF3 and ELOA in CRC. Fig. S8. DLGAP1-AS2 promoted the degradation of ELOA. Fig. S9. The function of ELOA andELOA as the downstream of DLGAP1-AS2. Fig. S10. The expression of LHPP and its relationship to ELOA in CRC. Fig. S11. CPSF2 and CSTF3 are associated with poor clinical outcomes in patients with CRC. Fig. S12. The effect of CPSF2 or CSTF3 on AKT. Tab. S1. Primer sequence. Tab. S2. siRNA sequences. Tab. S3. Relatedsequence information for plasmid construction. Tab. S4. The sequences for DLAGP1-AS2 and its deletion fragments used in vitro transcription. Tab. S5. Antibody information. Tab. S6. Relationships between DLGAP1-AS2 expression and clinical pathologic factors of the patients with CRC. Tab. S7. Relationships between ELOA expression and clinical pathologic factors of the patients with CRC. [file 12943_2022_1675_MOESM1_ESM.docx]

**Figure legends**


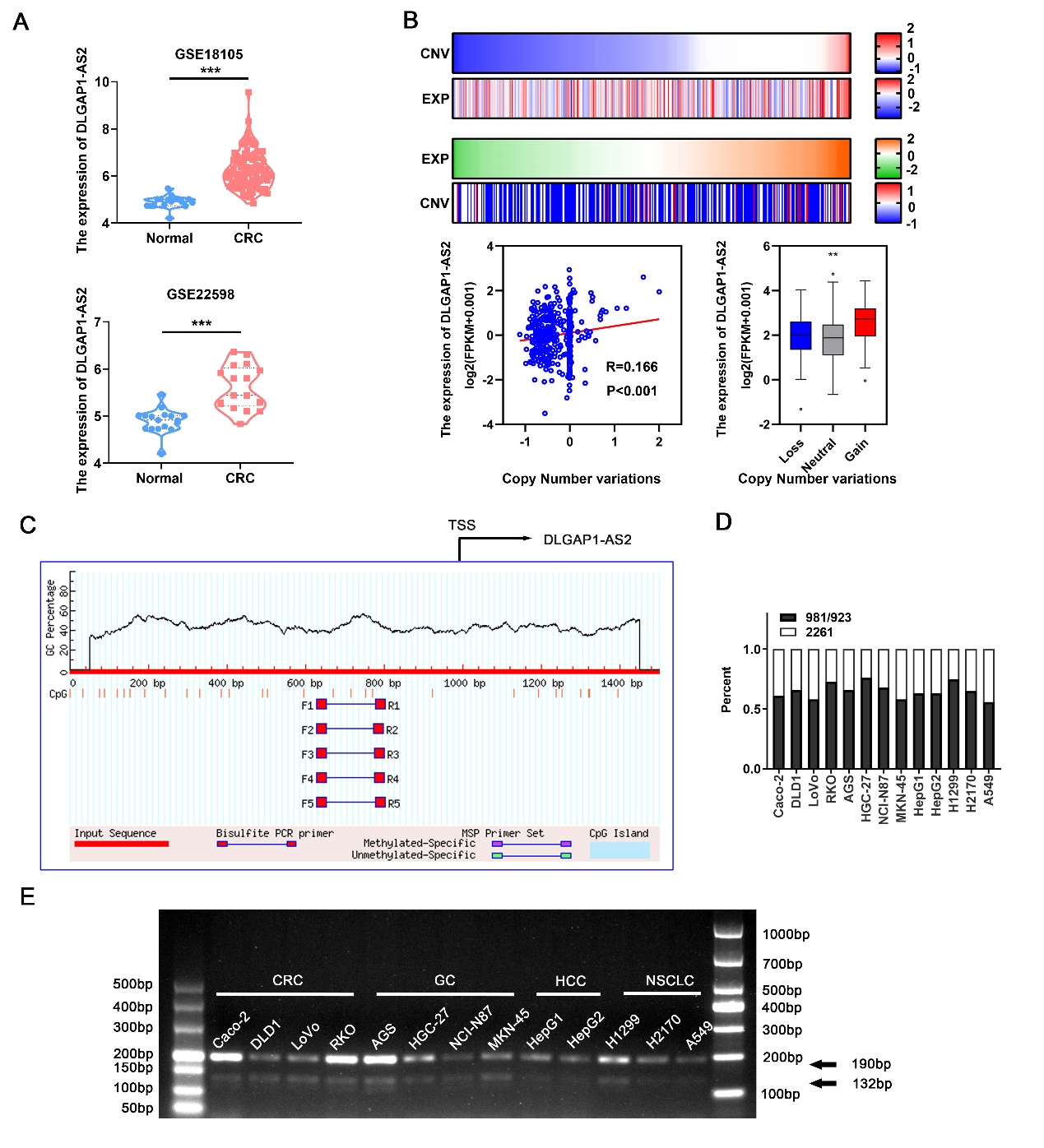


**Fig.S1 The expression and transcript analyses of DLGAP1-AS2.** (A) The expression of DLGAP1-AS2 in GEO datasets. (B) The copy number variations (CNVs) and expression of DLGAP1-AS2 in the TCGA CRC cohort. Correlation analyses between the CNV and expression of DLGAP1-AS2 in the TCGA CRC cohort. (C) CpG island prediction of DLGAP1-AS2 promoter using Methprimer (http://www.urogene.org/methprimer/). (D) The distribution of DLGAP1-AS2 transcripts in different types of cancer cells. (E) The relative levels of the two transcripts of DLGPA1-AS2 (981bp or 923 bp) in other types of cancer cells were detected with a semi-quantitative RT-PCR method. The bands with 190bp or 132 bp length represent the 981bp or 923 bp transcript of DLGAP1-AS2, respectively.


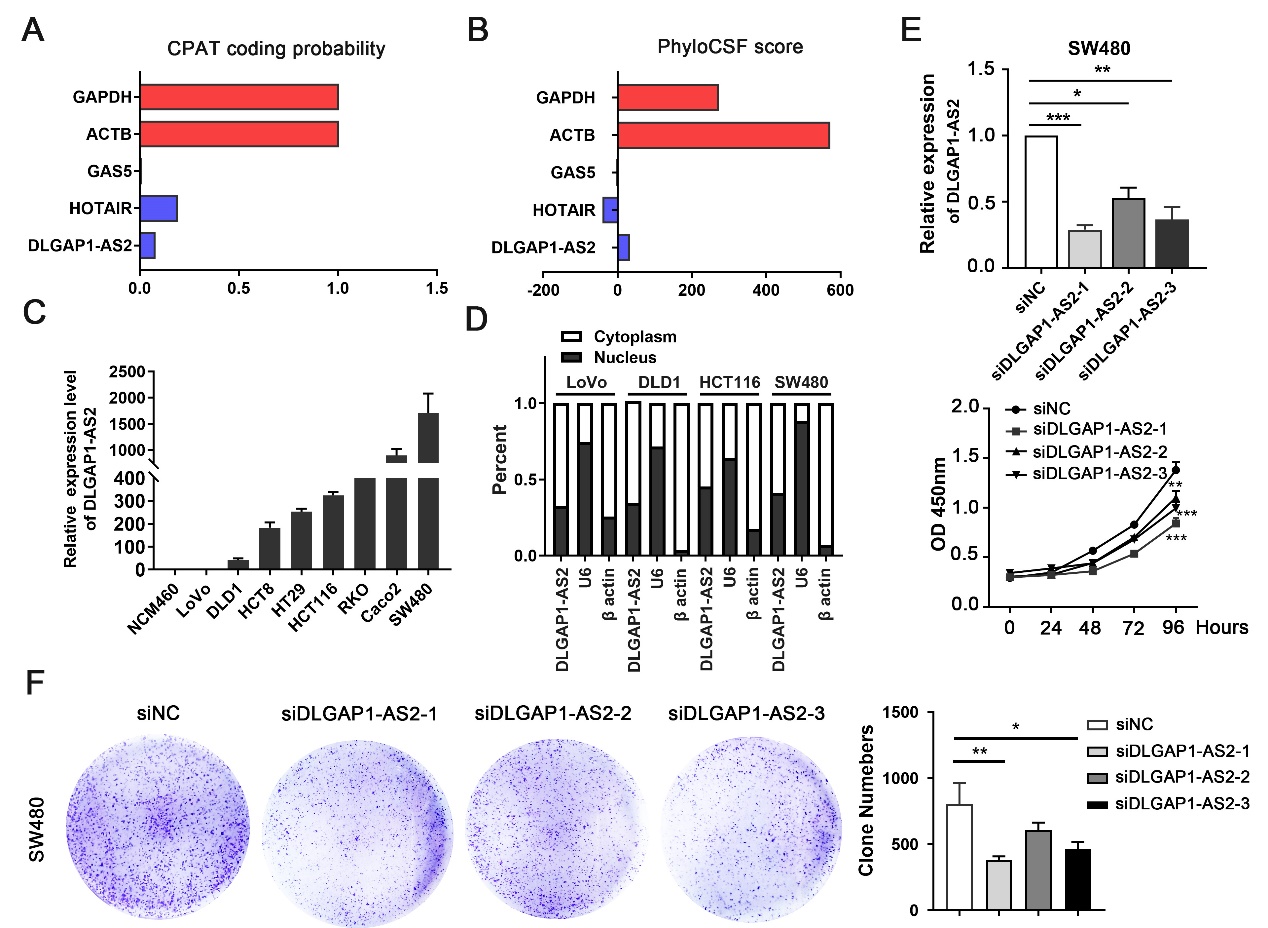


Fig.S2 Characterization of the protein-coding potential, subcellular localization and function for DLGAP1-AS2 in CRC. (A) The protein coding potential of DLGAP1-AS2 by using Coding Potential Assessment Tool (CPAT, <http://lilab.research.bcm.edu/cpat/>). GAPDH and ACTB served as the positive controls of coding genes, and GAS5 and HOTAIR served as the negative control of non-coding genes. (B) The protein coding potential of DLGAP1-AS2 predicted by the PhyloCSF software. (C) The RNA levels of DLGAP1-AS2 in CRC cell lines. (D) The distribution of DLGAP1-AS2 981bp transcript in CRC cell lines. β-actin served as the cytoplasmic internal control. U6 served as the nuclear internal control. (E) The effects of DLGAP1-AS2 knockdown on CRC cell proliferation. (F) The effects of DLGAP1-AS2 knockdown on CRC colony formation.


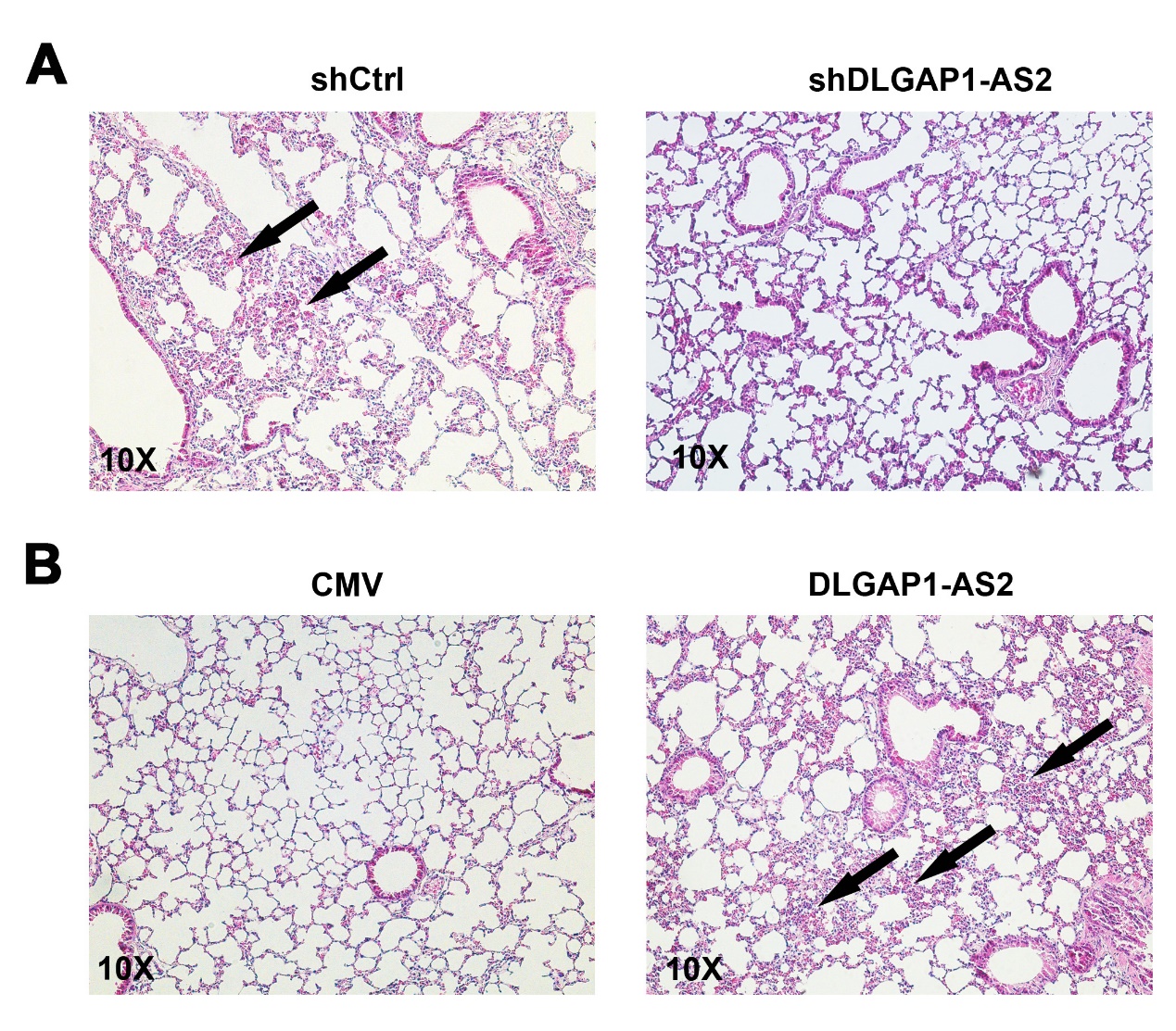


Fig.S3 HE staining for lung tissues **from a lung metastasis mouse model**.


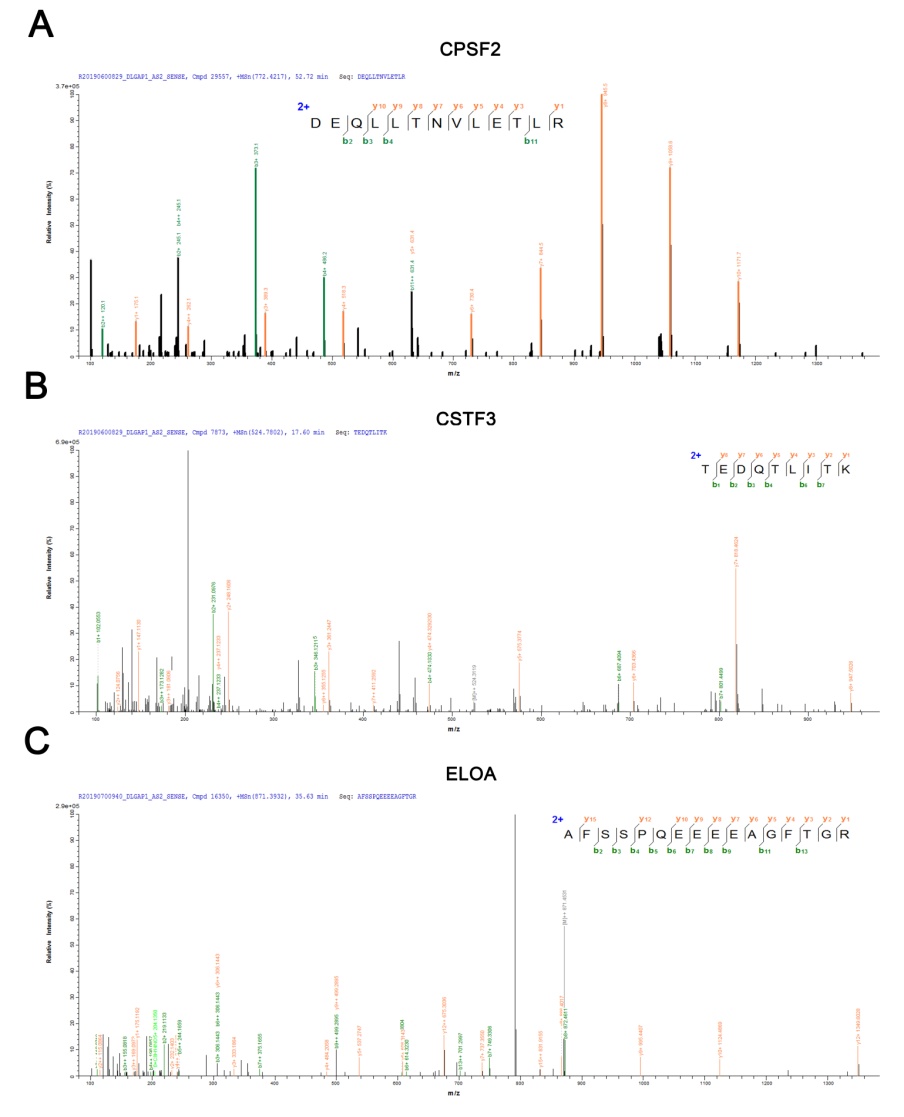


Fig.S4 **Mass spectrometry analyses in samples from** streptavidin pull down assays. (A-C) DLGAP1-AS2 Sense and Antisense RNA were biotinylated, transcribed in vitro and incubated with HCT116 cell lysates for RNA pull-down assays. After silver staining, the DLGAP1-AS2 sense-specific bands CPSF2, CSTF3 and ELOA were excised and analyzed using mass spectrometry.


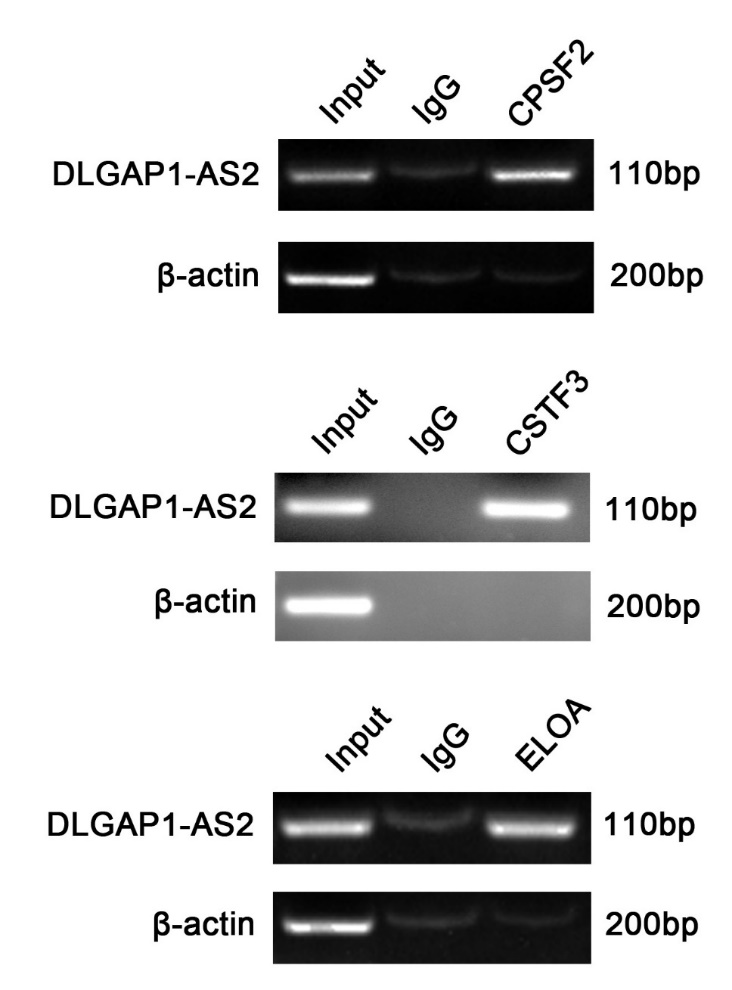


Fig.S5 RIP assays. RIP assays were performed to confirm the association of CPSF2, CSTF3 and ELOA with DLGAP1-AS2 using the indicated antibodies. Semi-quantitative RT-PCR were used to detect the enrichment of DLGAP1-AS2.


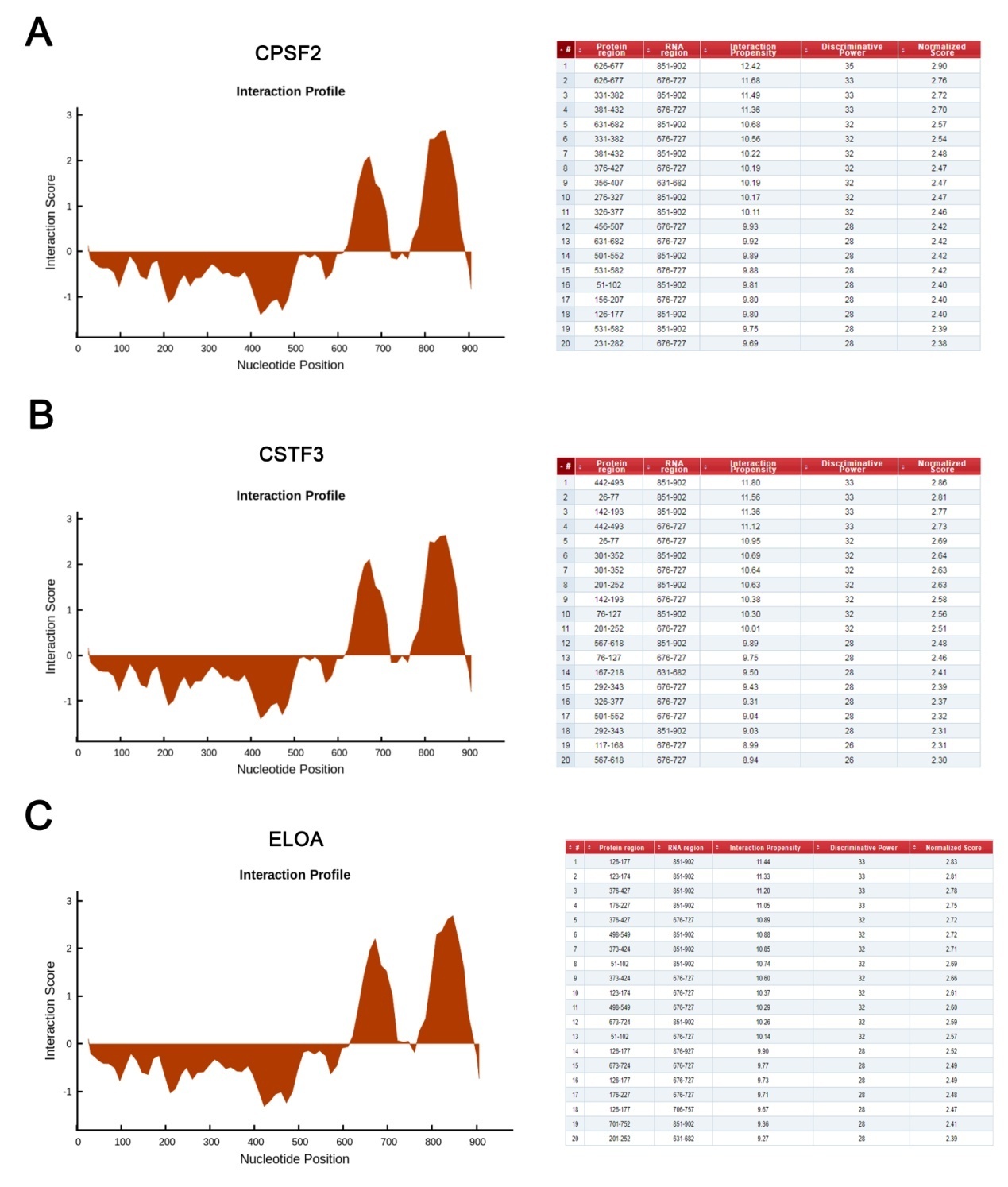


Fig.S6 Graphic illustration of predicted DLGAP1-AS2 secondary structure. (A-C) The software catRAPID predicted the interaction site between DLGAP1-AS2 and CPSF2, CSTF3 and ELOA.


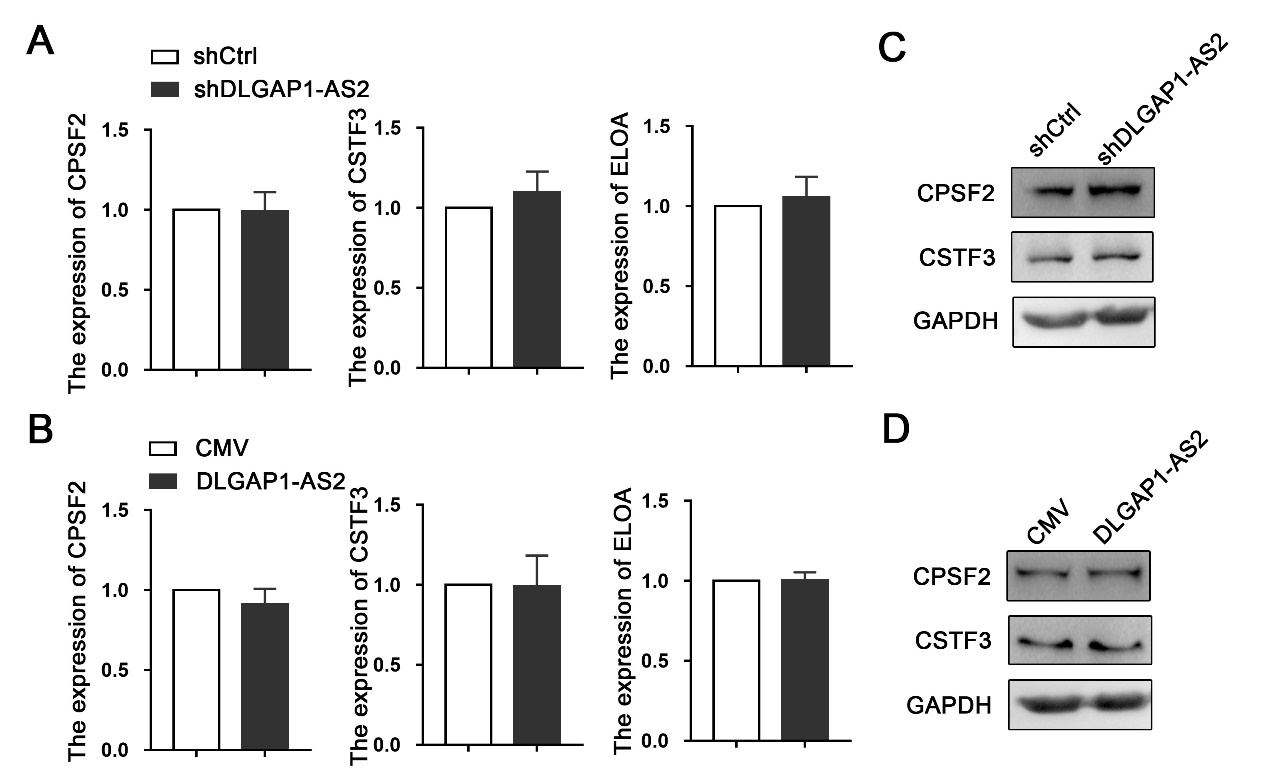


Fig.S7 The effect of DLGAP1 on CPSF2, CSTF3 and ELOA in CRC**.** (A) The mRNA levels of CPSF2, CSTF3 and ELOA were quantified by qRT-PCR in CRC cells with DLGAP1 knockdown.(B) The mRNA levels of CPSF2, CSTF3 and ELOA were quantified by qRT-PCR in CRC cells with DLGAP1-AS2 overexpression. (C-D) Immunoblotting for the protein levels of CPSF2 and CSTF3 after DLGAP1-AS2 knockdown or over-expression. GAPDH served as the internal control.


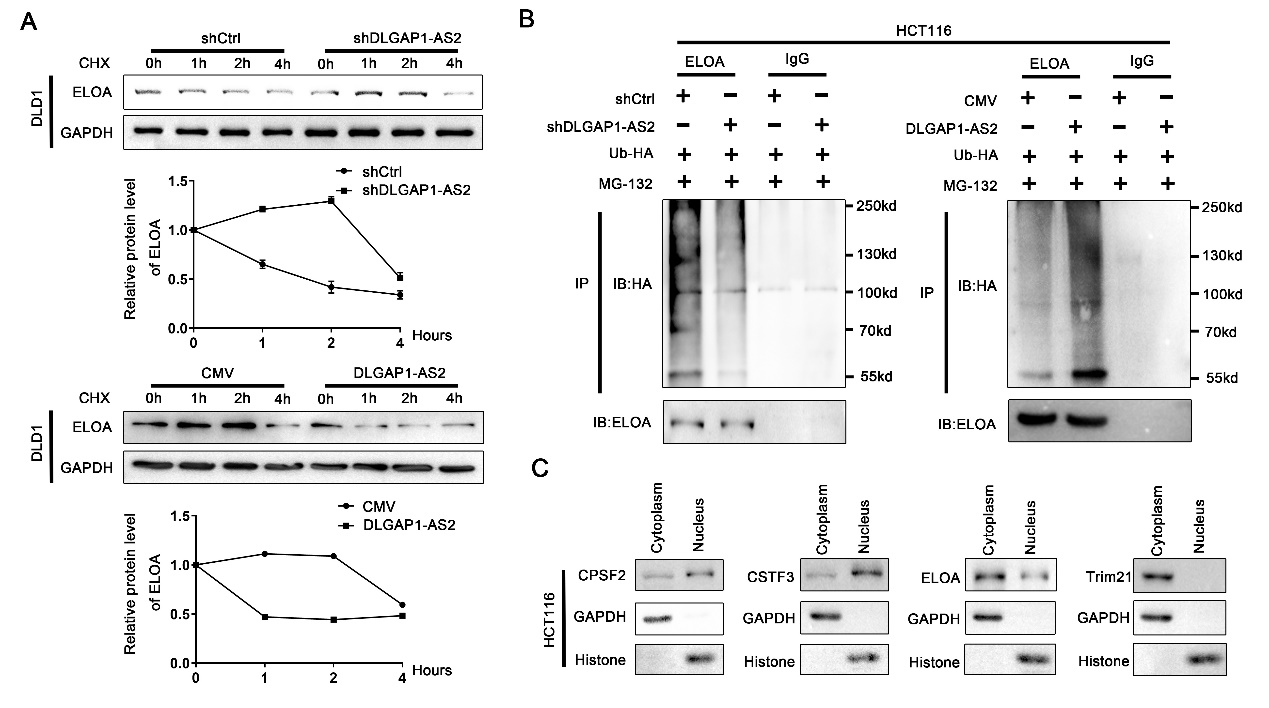


Fig.S8 DLGAP1-AS2 promoted the degradation of ELOA. (A) DLGAP1-AS2 promoted the degradation of ELOA in CRC cell. (B) DLGAP1-AS2 promoted the ubiquitination modification of endogenous ELOA. HCT116 cells transfected with HA-Ub or DLGAP1-AS2 plasmids were treated with MG132 for 4 hours before harvest. Ubiquitinated ELOA was measured by western blotting using an anti-HA antibody following the immunoprecipitation of ELOA. (C) The location of CPSF2, CSTF3, ELOA and Trim21 in HCT116 cells.


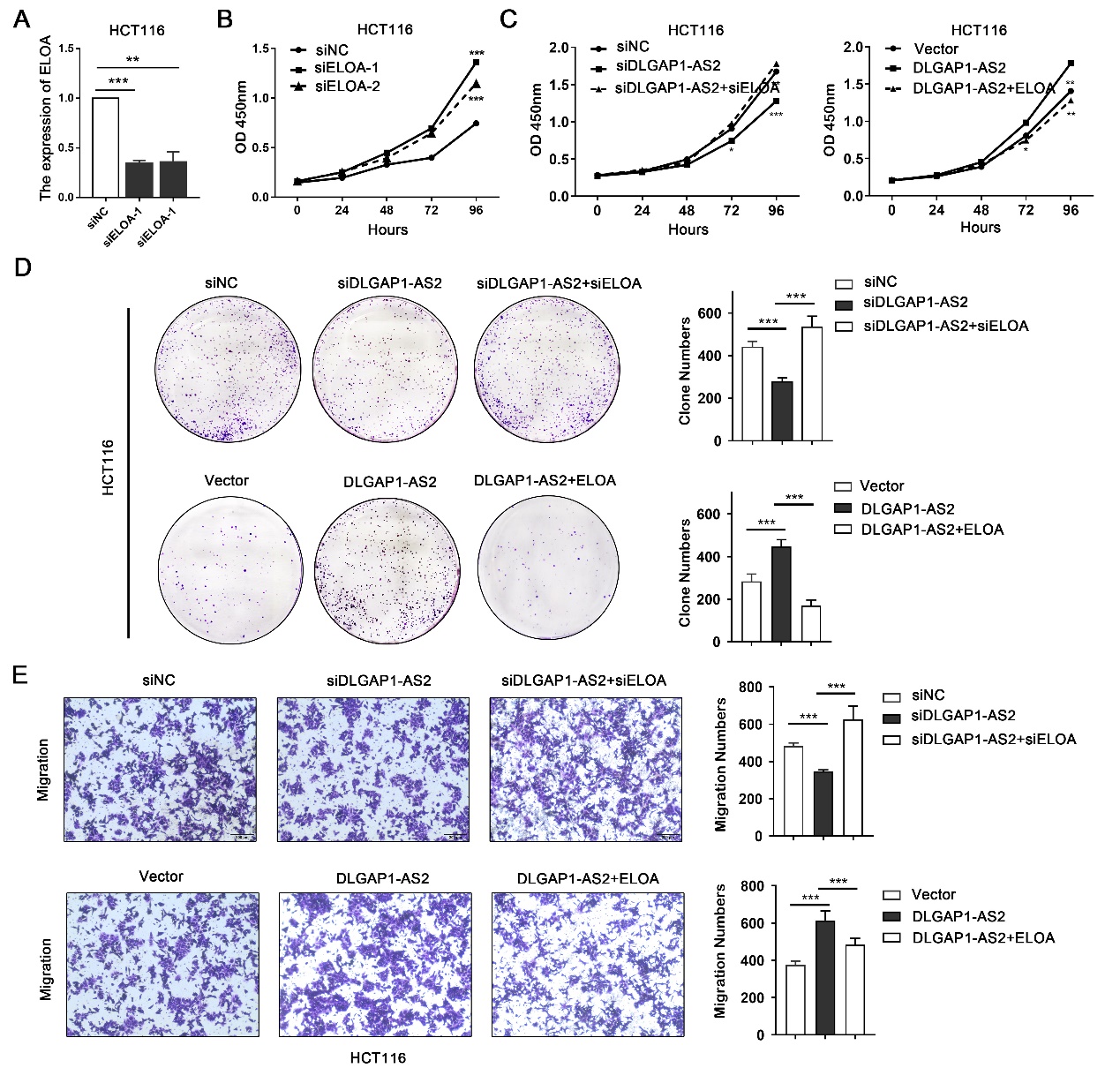


Fig.S9 The function of ELOA and ELOA as the downstream of DLGAP1-AS2. (A-B) siRNA-mediated silencing of ELOA promotes cell proliferation in HCT116 cells. (C) CCK-8 assays for HCT116 cells co-transfected with different siRNAs and/or plasmids. (D-E) Clone and Transwell assays for HCT116 cells transfected with DLGAP1-AS2 and ELOA.


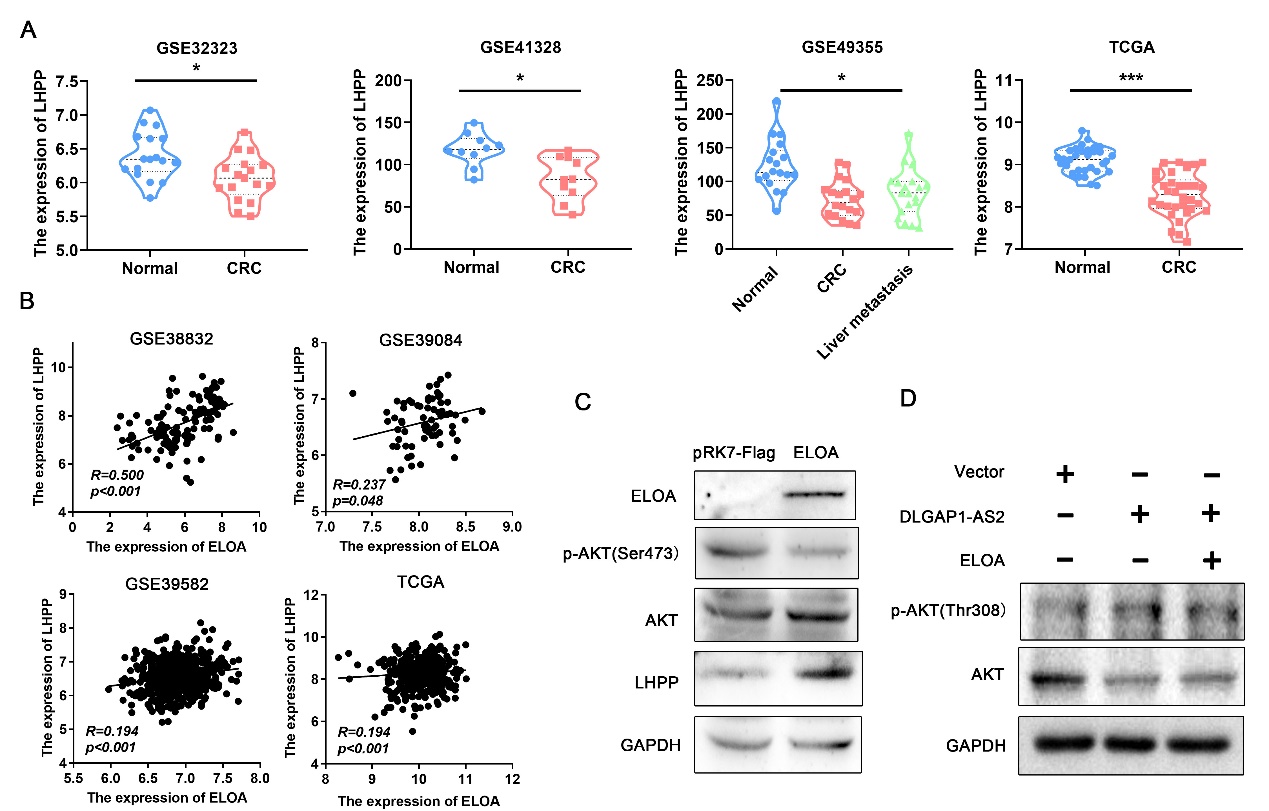


Fig.S10 The expression of LHPP and its relationship to ELOA in **CRC**. (A) Evaluation of LHPP expression in CRC tissues and their paired normal tissues based on the GEO and TCGA datasets. (B) The relationship between ELOA and LHPP. (C)The effect of ELOA on AKT pathway (pAKT at Ser473). (D) The effect of DLGAP1-AS2/ELOA on the AKT pathway (pAKT at Thr308).


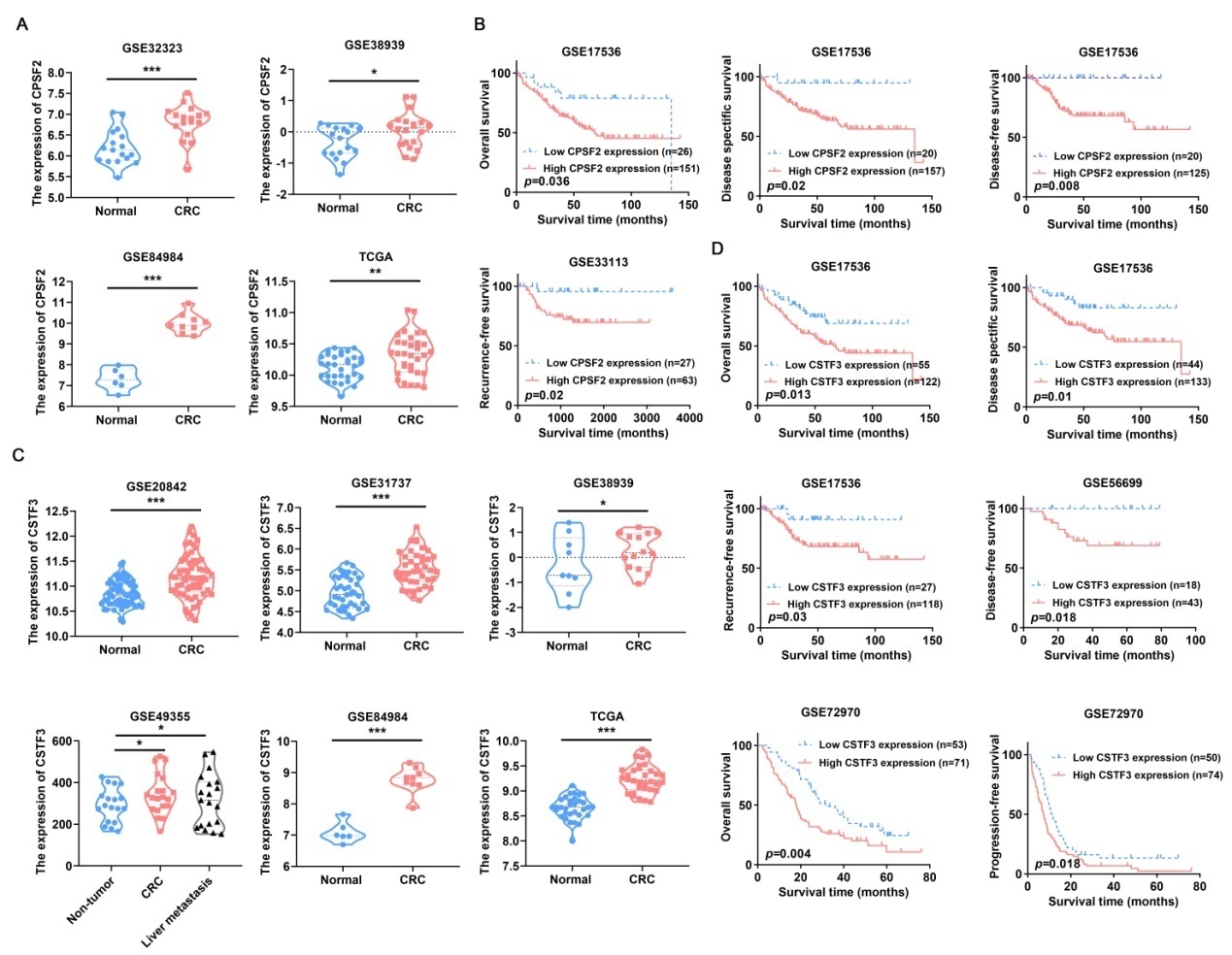


**Fig.S11 CPSF2 and CSTF3 are associated with poor clinical outcomes in patients with CRC.** (A) Evaluation of the mRNA expression of CPSF2 in CRC tissues and their paired normal tissues based on the GEO and TCGA datasets. (B) Kaplan-Meier plots of overall survival and disease-free survival versus CPSF2 from GEO datasets. (C) Evaluation of the expression of CSTF3 in CRC tissues and their paired normal tissues based on the GEO and TCGA datasets. (D) Kaplan-Meier plots of overall survival and disease-free survival versus CSTF3 from GEO datasets.


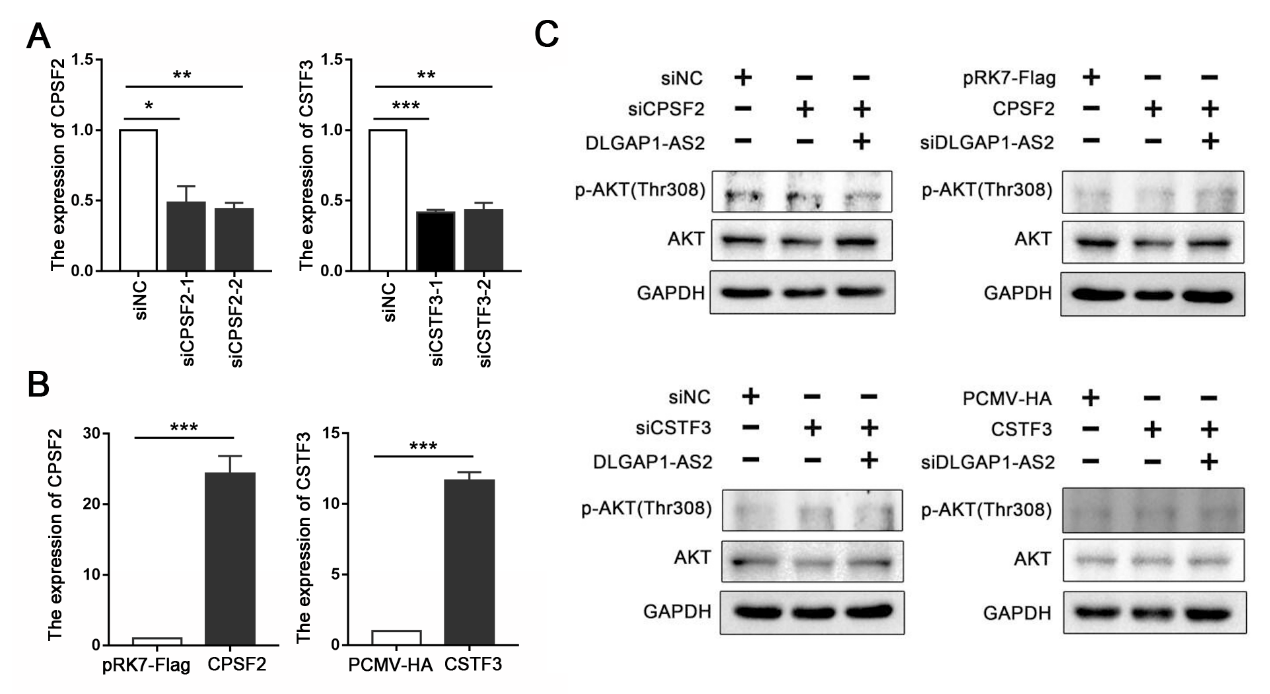


**Fig.S12 The effect of CPSF2 or CSTF3 on AKT.** (A) The inhibiting efficiencies of siRNAs in the mRNA expression of CPSF2 or CSTF3 in HCT116 cells. (B) The validation of CPSF2 and CSTF3 overexpression in HCT116 cells. (C) The effect of CPSF2 or CSTF3 on the phosphorylation of AKT at Thr308.

**Tab.S1 Primer sequence**

| Primer | sequence |
| --- | --- |
| DLGAP1-AS2(981)-F | GCAGTGAAGCATCAGAAAAGTATAGT |
| DLGAP1-AS2(981)-R | GACTTGATGTCAAAGCTGGGAG |
| DLGAP1-AS2(923)-F | GCAGTGAAGCATCAGAAAAGTATAGT |
| DLGAP1-AS2(923)-R | TCATATTTGAATTTTGTTGCATCAC |
| DLGAP1-AS2(2261)-F | TTTACTTTATTCTTTGGGCTTCTCTG |
| DLGAP1-AS2(2261)-R | CTAGTCATGTCATAGCAGTGAAGAAATA |
| CSTF3-F | TTATGAACGCCTTGTTGCCC |
| CSTF3-R | ATCACTACAGTGAATGCTGCAA |
| ELOA-F | TTCCAGCCAAAGCGAAAAGC |
| ELOA-R | TGCTGGTGCAAGGTCATCAT |
| β-actin-F | AGTGTGACGTGGACATCCGCAAAG |
| β-actin-R | ATCCACATCTGCTGGAAGGTGGAC |
| CPSF2-F | TGAAAGGTGAAGGCAGTCGT |
| CPSF2-R | AGGTAACCCGGGCTTTGATA |
| IL7-F | GGGTGAAGCCCAACCAACAA |
| IL7-R | CAGTGTTCTTTAGTGCCCATCAA |
| IL18-F | TGACCAAGGAAATCGGCCTC |
| IL18-R | GCCATACCTCTAGGCTGGCT |
| IL23A-F | CTCTGCTCCCTGATAGCCCT |
| IL23A-R | GGAGGCTGCGAAGGATTTTG |
| CD82-F | CAGTGATCCTGGGCTTCGG |
| CD82-R | AAGACATAGGCCCCCATCCT |
| CDK2-F | GCATCTTTGCTGAGATGGTGAC |
| CDK2-R | GCTTGTTAGGGTCGTAGTGC |
| LHPP-F | ATAACGCCTTCCAGGTGCTC |
| LHPP-R | ATCAGGCCAGAGGTCTCCTT |
| LHPP-1-F | TCCCACCCTCTTCCCTGTAAA |
| LHPP-1-R | CGGGACTCTGTGCTTTTCTGG |
| LHPP-2-F | CACCACATGGAGACAAACACT  ATAAGCAAGAAGACGAAGAAA |
| LHPP-2-R | ATAAGCAAGAAGACGAAGAAA |
| LHPP-3-F | TGCATTTTTCCTCCCAAGTGA |
| LHPP-3-R | CGGGTGGGCCTCTCCCAGTC |
| PDK1-F | GGCCAGGTGGACTTCTACG |
| PDK1-R | ACATTCTGGCTGGTGACAGG |
| CMPK2-F | ATGCCATAGCCACTGAGGTG |
| CMPK2-R | CGAAACACACTGTTGGCCTC |

**Tab.S2 siRNA sequences**

| siRNA | sequence |
| --- | --- |
| si-DLGAP1-AS2-1-sense  sense（5'-3'）  sense（5'-3'） | GCGCCUAAGAAAUGCCUGUTT |
| si-DLGAP1-AS2-1-antisense | ACAGGCAUUUCUUAGGCGCTT |
| si-DLGAP1-AS2-2-sense  sense（5'-3'）  sense（5'-3'） | CACAGACAAGACCCUUUCATT |
| si-DLGAP1-AS2-2-antisense | UGAAAGGGUCUUGUCUGUGTT |
| si-DLGAP1-AS2-3-sense  sense（5'-3'）  sense（5'-3'） | GGACUAUGCUUAACCCAUUTT |
| si-DLGAP1-AS2-3-antisense | AAUGGGUUAAGCAUAGUCCTT |
| si-ELOA-1633-sense | CCUUCCCUCGAGCUGAUAUTT |
| si-ELOA-1633-antisense | AUAUCAGCUCGAGGGAAGGTT |
| si-ELOA-1314-sense | GGAGACAGAUAUGGAGGAUTT |
| si-ELOA-1314-antisense | AUCCUCCAUAUCUGUCUCCTT |
| si-CPSF2-399-sense | GGAAGCAUGUUCACCAGAUTT |
| si-CPSF2-399-antisense | AUCUGGUGAACAUGCUUCCTT |
| si-CPSF2-509-sense | GCAACCAUUCCUGUUUAUATT |
| si-CPSF2-509-antisense | UAUAAACAGGAAUGGUUGCTT |
| si-CSTF3-1398-sense | GCAGCACUCAUGGAAUAUUTT |
| si-CSTF3-1398-antisense | AAUAUUCCAUGAGUGCUGCTT |
| si-CSTF3-240-sense | CCAUAUGACCUUGAUGCUUTT |
| si-CSTF3-240-antisense | AAGCAUCAAGGUCAUAUGGTT |
| Negative control-sense | UUCUCCGAACGUGUCACGUTT |
| Negative control-antisense | ACGUGACACGUUCGGAGAATT |

**Tab.S3 Related sequence information for plasmid construction**

| Primer | sequence |
| --- | --- |
| sh-DLGAP1-AS2-F | CCGGAAGCGCCTAAGAAATGCCTGTCTCGAGGTATAGCTTAACGTAGGCATTTTTTTG |
| sh-DLGAP1-AS2-R | AATTCAAAAAAAGCGCCTAAGAAATGCCTGTCTCGAGGTATAGCTTAACGTAGGCATT |
| sh-ELOA-F | CCGGAACCTTCCCTCGAGCTGATATCTCGAGATATCAGCTCGAGGGAAGGTTTTTTTG |
| sh-ELOA-R | AATTCAAAAAAACCTTCCCTCGAGCTGATATCTCGAGATATCAGCTCGAGGGAAGGTT |
| DLGAP1-AS2(981)-F | GTGAACCGTCAGATCGAATTCGTCTTTCAGGATGAATGCCATCC |
| DLGAP1-AS2(981)-R | TAATCCAGAGGTTGAGGATCCATTTTTTAAAGCAATTTTTTATTTTATTTTT |
| CPSF2-F | ctgcacctcggttctaagcttATGACGTCTATTATCAAATTAACTACCCT |
| CPSF2-R | gattgaattccccggggatccTTATACAATGGCATATTGTTCATATAAAAG |
| CPSF2-1-375-F | ctgcacctcggttctaagcttATGACGTCTATTATCAAATTAACTACCCT |
| CPSF2-1-375-R | gattgaattccccggggatccTTATTTTTCAGAAGGATTATCAATTAGGA |
| CPSF2-376-782-F | ctgcacctcggttctaagcttATTACAGAAATAGAGTTGAGGAAACGTG |
| CPSF2-376-782-F | acgacgatgacaagaagcttATGACGTCTATTATCAAATTAACTACCCT |
| CSTF3-F | gattacgcttctaggggatccGCCACCATGTCAGGAGACGG |
| CSTF3-R | ggttctagaatcgatgatatcCTACCGAATCCGCTTCTGCTG |
| CSTF3-1-374-F | gattacgcttctaggggatccGCCACCATGTCAGGAGACGG |
| CSTF3-1-374-R | ggttctagaatcgatgatatcCTAAGGGTCAATATCCTCAATTGCC |
| CSTF3-375-717-F | gattacgcttctaggggatccGCCACCACCTTGGTATATATCCA |
| CSTF3-375-717-R | ggttctagaatcgatgatatcCTACCGAATCCGCTTCTGCTG |
| ELOA-F | acgacgatgacaagaagcttATGCACGGAGGGCGGAGC |
| ELOA-R | attgaattccccggggatccTTATCGTCGGGAGAATCTGTTCTTG |
| ELOA-1-250-F | acgacgatgacaagaagcttATGCACGGAGGGCGGAGC |
| ELOA-1-250-R | attgaattccccggggatccTTAGGCCCCGAGTCTGTCCTGAA |
| ELOA-251-500-F | acgacgatgacaagaagcttATGAGCCAAGAACGACACCTG |
| ELOA-251-500-R | attgaattccccggggatccTTATCTCAGCTTGGCTAAATCAG |
| ELOA-501-798-F | acgacgatgacaagaagcttATGAAGGTGCCTGATGTGTTGC |
| ELOA-501-798-R | attgaattccccggggatccTTATCGTCGGGAGAATCTGTTCTTG |
| LHPP-positive-F | gcgtgctagcccgggctcgagTCCCACCCTCTTCCCTGTAAA |
| LHPP-positive-R | cagtaccggaatgccaagcttCGGGTGGGCCTCTCCCAG |
| LHPP-negative-F | gcgtgctagcccgggctcgagTCCTGCTGGAAGGACCAGC |
| LHPP-negative-R | cagtaccggaatgccaagcttCTCCCTCCCTCCATGGAGTC |
| h-Trim21-gRNA1 | CATGTTGGCTAGCTGTCGAT |
| h-Trim21-gRNA2 | TCATCTCAGAGCTAGATCGA |

**Tab.S4 The sequences for DLAGP1-AS2 and its deletion fragments used in the in vitro transcription**

| Primer | sequence |
| --- | --- |
| DLGAP1-AS2-sense-F | TAATACGACTCACTATAGGGAGAGTCTTTCAGGATGAATGC |
| DLGAP1-AS2-sense-R | ATTTTTTAAAGCAATTTTTTATTT |
| DLGAP1-AS2-antisense-F | GTCTTTCAGGATGAATGC |
| DLGAP1-AS2-antisense-R | TAATACGACTCACTATAGGGAGAATTTTTTAAAGCAATTTTTTATTT |
| DLGAP1-AS2-70-981-F | TAATACGACTCACTATAGGGAGAACACAGACAAGACCCTTTCAAT |
| DLGAP1-AS2-70-981-R | ATTTTTTAAAGCAATTTTTTATTTTAT |
| DLGAP1-AS2-1-379-F | TAATACGACTCACTATAGGGAGAGTCTTTCAGGATGAATGCC |
| DLGAP1-AS2-1-379-R | TTCTTTACACATAAAGTTGGC |
| DLGAP1-AS2-380-981-F | TAATACGACTCACTATAGGGAGAGCTAACTCCTGCCAACATC |
| DLGAP1-AS2-380-981-R | ATTTTTTAAAGCAATTTTTTATTT |
| DLGAP1-AS2-1-620-F | TAATACGACTCACTATAGGGAGAGTCTTTCAGGATGAATGC |
| DLGAP1-AS2-1-620-R | AATCCCATTACGTGGC |
| DLGAP1-AS2-621-981-F | TAATACGACTCACTATAGGGAGAACGGATTATGTGATGCAACA |
| DLGAP1-AS2-621-981-R | ATTTTTTAAAGCAATTTTTTATTT |

**Tab.S5 Antibody information**

| **Antibody** | **Company** | **Cat No.** |
| --- | --- | --- |
| ELOA mouse antibody | Santa Cruz Biotechnology | sc-373811 |
| ELOA rabbit antibody | proteintech | 10502-1-AP |
| CstF-77 antibody | Santa Cruz Biotechnology | sc-376575 |
| CPSF2 antibody | proteintech | 17739-1-AP |
| Trim21 | proteintech | 12108-1-AP |
| Flag tag Antibody Mouse Monoclonal | sigma | MA1-91878 |
| Flag tag Antibody Rabbit Polyclonal | proteintech | 20543-1-AP |
| HA-Tag Rabbit mAb | CST | CST 3724 |
| Myc-Tag Rabbit mAb | abmart | M2002L |
| GAPDH Antibody | ABclonal | AC033 |
| LHPP | proteintech | 15759-1-AP |
| AKT | proteintech | 10176-2-AP |
| p-AKT | proteintech | 66444-1-Ig |
| p-AKT(Thr308) | CST | 13038T |

**Tab.S6 Relationships between DLGAP1-AS2 expression and clinical pathologic factors of the patients with CRC**

| **Characteristic** | **Number**  **(n=111)** | **P_a_ value** | **P_b_ value** | **Overall Survival**  ***HR(95%CI)_b_*** |
| --- | --- | --- | --- | --- |
| **Age(years)** |  |  |  |  |
| ＜60 | 62 | 0.960 | 0.234 | 1.491（0.772-2.879） |
| ≥60 | 49 |  |  |  |
| **Gender** |  |  |  |  |
| Male | 66 | 0.404 | 0.224 | 0.651（0.325-1.302） |
| Female | 45 |  |  |  |
| **Size** |  |  |  |  |
| ＜5 | 69 | 0.576 | 0.202 | 1.532（0.796,2.948） |
| ≥5 | 42 |  |  |  |
| **Location** |  |  |  |  |
| Colon | 57 | 0.741 | 0.920 | 1.034（0.538,1.989） |
| Rectum | 54 |  |  |  |
| **Tumor differentiation** |  |  |  |  |
| High | 5 | 0.903 | **0.01** | 2.423（1.239,4.738） |
| Medium | 87 |  |  |  |
| Poor | 19 |  |  |  |
| **TNM** |  |  |  |  |
| 1 | 18 | **0.01** | **＜0.001** | 4.429（2.682,7.312） |
| 2 | 32 |  |  |  |
| 3 | 50 |  |  |  |
| 4 | 11 |  |  |  |
| **Lymph node metastasis** |  |  |  |  |
| 0 | 51 | **0.031** | **＜0.001** | 4.751（2.838,7.955） |
| 1 | 30 |  |  |  |
| 2 | 30 |  |  |  |
| **Gene expression** |  |  | ***P_c_*** | ***HR(95%CI)_c_*** |
| Low | 31 | **0.021** | **0.030** | 3.967  (1.296,12.139) |
| High | 80 |  |  |  |

**Tab.S7 Relationships between ELOA expression and clinical pathologic factors of the patients with CRC**

| **Characteristic** | **Number**  **(n=146)** | **Low Expression** | **High**  **Expression** | **P_a_ value** | **P_b_ value** | **Overall Survival**  ***HR(95%CI)_b_*** |
| --- | --- | --- | --- | --- | --- | --- |
| **Age(years)** |  |  |  |  |  |  |
| ＜60 | 68 | 45 | 23 | 0.414 | **0.013** | 1.673（1.115-2.511） |
| ≥60 | 78 | 54 | 24 |  |  |  |
| **Gender** |  |  |  |  |  |  |
| Male | 81 | 55 | 26 | 0.559 | 0.629 | 0.649（1.152-2.044） |
| Female | 65 | 44 | 21 |  |  |  |
| **Size** |  |  |  |  |  |  |
| ＜5 | 78 | 51 | 27 | 0.576 | 0.679 | 0.922（0.628-1.354） |
| ≥5 | 67 | 47 | 20 |  |  |  |
| **Location** |  |  |  |  |  |  |
| Colon | 62 | 44 | 18 | 0.302 | 0.009 | 0.527（0.326-0.851） |
| Rectum | 84 | 52 | 29 |  |  |  |
| **Tumor**  **differentiation** |  |  |  |  |  |  |
| High | 17 | 9 | 8 | 0.298 | 0.204 | 1.283（0.874-1.883） |
| Medium | 108 | 74 | 34 |  |  |  |
| Poor | 21 | 16 | 5 |  |  |  |
| **TNM** |  |  |  |  |  |  |
| I/II | 96 | 58 | 38 | **0.007** | **＜0.001** | 2.864（1.932-4.248） |
| III/IV | 50 | 41 | 9 |  |  |  |
| **Expression** |  |  |  |  | ***P_c_*** | ***HR(95%CI)_c_*** |
| Low | 99 |  |  | **0.021** | 0.058 | 0.490 (0.234-1.025) |
| High | 47 |  |  |  |  |  |
